# Serine-Driven Metabolic Plasticity Drives Adaptive Resilience in Pancreatic Cancer Cells

**DOI:** 10.3390/antiox14070833

**Published:** 2025-07-07

**Authors:** Marcella Bonanomi, Sara Mallia, Mariafrancesca Scalise, Tecla Aramini, Federica Baldassari, Elisa Brivio, Federica Conte, Alessia Lo Dico, Matteo Bonas, Danilo Porro, Cesare Indiveri, Christian M. Metallo, Daniela Gaglio

**Affiliations:** 1 Institute of Molecular Bioimaging and Complex Biological Systems (IBSBC), National Research Council (CNR), 20054 Segrate, MI, Italy; marcella.bonanomi@cnr.it (M.B.); sara.mallia@cnr.it (S.M.); teclaaramini@cnr.it (T.A.); federica.baldassari@cnr.it (F.B.); elisa.brivio1@unimib.it (E.B.); alessia.lodico@cnr.it (A.L.D.); 2 National Biodiversity Future Center (NBFC), 90133 Palermo, PA, Italy; danilo.porro@unimib.it; 3 Unit of Biochemistry and Molecular Biotechnology, Department DiBEST (Biologia Ecologia Scienze della Terra), University of Calabria, 87036 Arcavacata di Rende, CS, Italy; mariafrancesca.scalise@unical.it (M.S.); cesare.indiveri@unical.it (C.I.); 4 Department of Biotechnology and Bioscience, University of Milano-Bicocca, 20126 Milano, MI, Italy; matteo.bonas@unimib.it; 5 Institute for Systems Analysis and Computer Science “Antonio Ruberti” (IASI), National Research Council (CNR), 00185 Rome, RM, Italy; federica.conte@iasi.cnr.it; 6 Institute of Biomembranes, Bioenergetics and Molecular Biotechnologies (IBIOM), National Research Council (CNR), 70126 Bari, BA, Italy; 7 Molecular and Cellular Biology Laboratory, Salk Institute for Biological Studies, La Jolla, CA 92037, USA; metallo@salk.edu

**Keywords:** pancreatic cancer, drug resistance, serine metabolism, metabolic rewiring, targeted therapy

## Abstract

Pancreatic cancer is one of the most lethal malignancies, in part due to its profound metabolic adaptability, which underlies drug resistance and therapeutic failure. This study explores the metabolic rewiring associated with resistance to treatment using a systems metabolomics approach. Exposure to the redox-disrupting agent erastin revealed key metabolic vulnerabilities but failed to produce lasting growth suppression. Combinatorial treatments with methotrexate or alpelisib significantly impaired proliferation and triggered marked metabolic shifts. Systems-level analyses identified serine metabolism as a central adaptive pathway in resilient cells. Metabolic tracing and gene expression profiling showed increased de novo serine biosynthesis and uptake, supporting redox homeostasis, biosynthetic activity, and epigenetic regulation. Notably, cells that resumed growth after drug withdrawal exhibited transcriptional reprogramming involving serine-driven pathways, along with elevated expression of genes linked to survival, proliferation, and migration. These findings establish serine metabolism as a functional biomarker of metabolic plasticity and adaptive resilience in pancreatic cancer, suggesting that targeting this adaptive axis may enhance therapeutic efficacy.

## 1. Introduction

The ability of cancer cells to adapt their metabolic processes in response to changes in nutrient availability and energy demands is a key driver of their growth, proliferation, and aggressiveness [1]. Pancreatic ductal adenocarcinoma (PDAC), one of the most common and aggressive pancreatic cancers, exhibits profound metabolic heterogeneity and lacks reliable early biomarkers, contributing to poor treatment outcomes [2]. This heterogeneity can be observed at the histological and molecular levels but is most pronounced in metabolic adaptations. Increasing evidence suggests that metabolic rewiring plays a pivotal role in PDAC progression and therapy resistance. Dysregulated glucose, glutamine, and lipid metabolism, along with metabolic interactions between tumor and stromal cells, create a microenvironment that fuels cancer progression [3,4,5].

PDAC relies heavily on oncogenic KRAS signaling to drive metabolic rewiring. Nearly 90% of PDAC cases harbor KRAS mutations, which activate downstream pathways that support tumor growth, migration, and metabolic adaptation [6]. Given the central role of KRAS in PDAC pathophysiology, targeting both RAS-driven signaling and metabolic vulnerabilities is emerging as a promising therapeutic strategy [3].

A promising strategy could involve erastin, a well-characterized RAS inhibitor known for inducing synthetic lethality in *KRAS*-mutant cancer cells by triggering oxidative stress [7]. Beyond its effects on RAS signaling, erastin also disrupts metabolic homeostasis by inhibiting system xCT, a critical transporter responsible for cystine uptake and glutathione (GSH) synthesis [8,9,10]. GSH is essential for maintaining redox balance and shielding cancer cells from oxidative damage. Erastin-driven GSH depletion exposes PDAC cells to excessive oxidative stress, compromising their survival [11]. Additionally, erastin, by disrupting mitochondrial function through its interaction with the voltage-dependent anion channel (VDAC), impairing energy production, amplifying oxidative stress, and inducing ferroptotic cell death, becomes a powerful tool for investigating metabolic vulnerabilities in PDAC [12,13]. While preliminary studies suggest that erastin effectively inhibits PDAC cell growth [14,15,16], the underlying metabolic adaptations and resistance mechanisms remain poorly understood [3,17].

To address these gaps, we conducted a comprehensive investigation into the metabolic rewiring associated with erastin treatment in PDAC. First, we characterized the metabolic signature of erastin across a panel of eight PDAC cell lines. Next, we identified FDA-approved drugs that could enhance the effects of erastin by targeting redox metabolism. Finally, we explored metabolic alterations associated with reduced drug sensitivity, shedding light on adaptive survival strategies in PDAC. The observed metabolic plasticity under drug treatments suggests that resistance mechanisms may emerge as a downstream consequence of adaptive metabolic response.

By unraveling drug-induced metabolic adaptations, this multistep approach deepens our understanding of pancreatic cancer’s metabolic landscape, offering promising directions for precision medicine and a new approach for selecting therapeutic targets.

## 2. Materials and Methods

### 2.1. Cell Culture

AsPC-1, BxPC-3, Capan-2, CFPAC-1, HPAF-2, PANC-1, Panc 10.05, and SW1990 pancreatic cancer (PDAC) cells were grown in Roswell Park Memorial Institute (RPMI) 1640 supplemented with 10% fetal bovine serum (FBS) and 2 mM L-glutamine. All media were supplemented with 100 U/mL penicillin and 100 μg/mL streptomycin. Cells were grown at 37 °C in a 5% CO_2_ incubator. Cell lines were obtained from the American Type Culture Collection (ATCC) (LGC Chemicals Standard, Teddington, UK). Cell culture reagents were purchased from Life Technologies (Waltham, MA, USA), except for RPMI 1640 without serine obtained from USBiological (Salem, MA, USA).

### 2.2. Cell Proliferation Analysis

For proliferation curves under nutrient deprivation conditions, cells were plated in 6-well plates and the medium was replaced after 24 h with either normal growth medium, low-glutamine medium (0.5 mM Gln), or low-glucose medium (1 mM Glc). Cells were collected after 24, 48, 72, and 144 h and counted using a Countess^®^ II FL Automated Cell Counter (Thermo Fisher Scientific, Waltham, MA, USA). For dose–response curves, the Cell Counting Kit-8 (CCK-8) assay (Sigma-Aldrich, St Louis, MO, USA) was used to assess the cytotoxicity. Cells were plated in 96-well plates and after 48 h of drug exposure at the indicated concentrations, 10 μL of CCK-8 were added to each well, followed by incubation for 2 h at 37 °C. Absorbance was read at 450 nm in a Victor 3 microplate reader (PerkinElmer, Shelton, CT, USA). The drugs tested are erastin (MedChemExpress, Monmouth Junction, NJ, USA), alpelisib (MedChemExpress), methotrexate (SelleckChem, Houston, TX, USA), resveratrol (Sigma-Aldrich), and everolimus (Cayman Chemical, Ann Arbor, MI, USA). Cell viability was expressed as a percentage of control cells. For long proliferation curves in the presence of the drugs, cells were plated in 12-well plates and after 24 h the drugs were added at the indicated concentrations. Cells were collected and counted after 24, 48, 72, and 144 h. For proliferation curves with drug withdrawal, cells were plated in 6-well plates and treated with the indicated combination of drugs for 72 h, then the medium was replaced with fresh medium without drugs for another 72 h. After that, the cells were placed in the presence or absence of the drugs for the next 72 h. Cells were collected and counted at 72, 144, and 216 h.

### 2.3. Metabolite Extraction from Cell Culture

For untargeted experiments, cells were plated in 6-well plates with normal growth medium; the culture medium was replaced after 24 h with complete fresh medium in the presence or the absence of treatments and cells were then incubated for 48 h. For labeling experiments, cells were incubated for 48 h in fresh media supplemented with 11 mM [U-^13^C_6_]glucose, 2 mM [U-^13^C_5_]glutamine, or 0.285 mM [U-^13^C_3_]serine (all stable isotopes are purchased by Cambridge Isotope Laboratories, Tewksbury, MA, USA) in the presence or the absence of treatments. Metabolite extraction for GC-MS analysis was performed as described previously [18]. Briefly, cells were quenched with 1:1 ice-cold methanol–water and collected by scraping. After sonication, one volume of chloroform was added, and cells were vortexed at 4 °C for 20 min. Metabolite extraction for LC-MS analysis was performed as described previously [19]. Briefly, cells were quenched with an ice-cold solution of 70:30 acetonitrile–water, placed at 80 °C for 10 min, and then collected by scraping and then sonicated. For both extractions, samples were centrifuged at 12,000× *g* for 10 min and supernatant aqueous phases were collected in a glass insert and dried in a centrifugal vacuum concentrator (Concentrator plus/Vacufuge plus, Eppendorf, Hamburg, Germany) at 30 °C for about 2.5 h.

### 2.4. Metabolites Quantification in the Media Samples

Absolute quantification of glucose, lactate, glutamine, and glutamate in spent media was determined enzymatically using the YSI2950 bioanalyzer (YSI Incorporated, Yellow Springs, OH, USA). Media collected from experiments were thawed and centrifuged at 2000× *g* for 5 min before analysis. The YSI bioanalyzer employed enzyme-based biosensors for measuring glucose, lactate, glutamate, and glutamine concentrations. The biosensors used oxidase-containing membranes for oxidizing substrates, releasing hydrogen peroxide. The hydrogen peroxide was detected amperometrically on a platinum electrode surface. The current flow at the electrode was directly proportional to the hydrogen peroxide concentration and hence to the substrate concentration. Glucose, lactate, glutamine, and glutamate standard solutions were used to calibrate the instrument. Glucose and glutamine consumption as well as lactate and glutamate release were calculated as follows: consumption = mmol/L of compound in fresh complete media − mmol/L of compound in cultured media and release = mmol/L of compound in cultured media − mmol/L of compound in fresh complete media. The rates were reported as mmol/L per 10^5^ cells.

### 2.5. Metabolite Extraction from Media Samples

Media collected from experiments were thawed and centrifuged at 2000× *g* for 5 min. A total of 400 µL of ice-cold 80:20 methanol–water were added to 50 µL of medium. Samples were placed in a thermoshaker for 10 min at 2000 rpm at 4 °C and then centrifuged at 12,000× *g* for 10 min. Supernatants were collected in a glass insert and dried in a centrifugal vacuum concentrator (Eppendorf) at 30 °C for about 2.5 h.

### 2.6. GC-MS Metabolic Profiling

Dried polar metabolites were dissolved in 60 µL of 2% methoxyamine hydrochloride in pyridine (Thermo Fisher Scientific) and held at 40 °C for 6 h. After the reaction, 90 µL of MTBSTFA +1% TBDMCS (Thermo Fisher Scientific) were added and samples were incubated at 60 °C for 1 h. Derivatized samples were analyzed by GC-MS using a DB-35MS column (30 m × 0.25 mm × 0.25 μm) installed in an Agilent Intuvo 9000 gas chromatograph (GC) (Agilent Technologies, Santa Clara, CA, USA) interfaced with a 5977B mass spectrometer (MS) (Agilent Technologies, Santa Clara, CA, USA) operating under electron impact (EI) ionization at 70 eV. Samples (2 µL) were injected in splitless mode at 270 °C, using helium as the carrier gas at a flow rate of 1 mL/min. The GC oven temperature was held at 100 °C for 3 min and increased to 300 °C at 3.5 °C/min. For untargeted experiment, GC/MS data processing was performed using Agilent MassHunter Quantitative Analysis (ver B 07.01) software using NIST mass spectral library. Relative metabolite abundance was carried out after normalization to internal standard d27 Myristic acid. For labeling experiments, data were preprocessed using the OpenChrom 1.5.16 software package to convert raw data (.D) in NetCDF format. Mass isotopologue distributions (MIDs) were determined using Matlab R2011b by integrating metabolite ion fragments and correcting for natural abundance using in-house algorithms adapted from [20].

### 2.7. LC-MS Metabolic Profiling

Dried samples were resuspended with 150 µL H_2_O and then analyzed in a UHPLC-QTOF mass spectrometer (Agilent Technologies). LC separation was performed using an Agilent 1290 Infinity UHPLC system and an InfintyLab Poroshell 120 PFP column (2.1 × 100 mm, 2.7 μm; Agilent Technologies). Mobile phase A was water with 0.1% formic acid. Mobile phase B was acetonitrile with 0.1% formic acid. The injection volume was 15 μL and LC gradient conditions were as follows: 0 min: 100% A; 2 min: 100% A; 4 min: 99% A; 10 min: 98% A; 11 min: 70% A; 15 min: 70% A; 16 min: 100% A with 5 min of post-run. The flow rate was 0.2 mL/min and the column temperature was 35 °C. MS detection was performed using an Agilent 6550 iFunnel Q-TOF mass spectrometer (Agilent Technologies) with a Dual JetStream source operating in negative ionization mode. MS parameters were gas temp: 285 °C; gas flow: 14 L/min; nebulizer pressure: 45 psig; sheath gas temp: 330 °C; sheath gas flow: 12 L/min; VCap: 3700 V; Fragmentor: 175 V; Skimmer: 65 V; Octopole RF: 750 V. Active reference mass correction was conducted through a second nebulizer using masses with *m*/*z*: 112.9855 and 1033.9881. Data were acquired from *m*/*z* 60–1050. Data analysis and isotopic natural abundance correction were performed with MassHunter ProFinder (version 10.0) and MassHunter VistaFlux software (version 10.0.2) (Agilent Technologies) [21]. Data preprocessing was performed using the Batch Targeted Feature Extraction algorithm and Agile 2 algorithm. This software assigned identities to metabolites by searching against an in-house compound database built with Agilent PCDL Manager (version B.08.00) based on the metabolite formula and its corresponding retention time with a score > 75. Peak areas obtained were normalized for protein content for each sample.

### 2.8. Metabolomics Statistical Data Analysis

Metabolomics data were analyzed using Mass Profiler Professional 15.1 software (Agilent Technologies). Raw data were transformed in log2 scale and normalized using Pareto scaling. Data were then filtered, retaining in the analysis the entities that were at least present in 80.0 percent of the samples in one condition. Statistical analysis was performed by applying an unpaired *t*-test or one-way ANOVA analysis with a *p*-value cut-off of 0.05. Data visualization of significant entities was performed using a hierarchical clustering algorithm.

### 2.9. Oxygen Consumption Rate Analysis

The cellular oxygen consumption rate (OCR) was measured by the Seahorse XF extracellular flux analyzer (Seahorse Bioscience Inc., North Billerica, MA, USA) according to the manufacturer’s instructions. Briefly, cells were seeded in Seahorse XF 24-well assay plates and treated according to the experiment. On the day of the assay, the medium was washed and replaced with a pre-warmed assay medium (non-buffered DMEM supplemented with 1 mM sodium pyruvate, 11 mM glucose, and 2 mM glutamine, pH 7.4), and incubated in a non-CO_2_ incubator at 37 °C for 60 min. Basal levels of OCR were recorded, followed by the mitochondrial stress test using the inhibitor of ATP synthase oligomycin (1 μM), the uncoupler FCCP (1μM), or the electron transport inhibitor rotenone/antimycin A (0.5 μM). The following formulas were used to calculate respiratory parameters: Basal Respiration = OCR_basal_ − OCR_rot/ant_. Maximal Respiration = OCR_FCCP_ − OCR_rot/ant_. Spare respiratory capacity = Maximal respiration—Basal Respiration. ATP mitochondrial production: OCR_basal_ − OCR_oligo_.

### 2.10. ROS Levels Measurement

Total ROS levels and Mitochondrial ROS levels were measured using a Dichloro-dihydro-fluoresceine-diacetate (DCFDA) Cellular ROS Detection Assay Kit (Abcam, Cambridge, UK) and MitoSOX™ Red Mitochondrial Superoxide Indicator (Thermo Fisher Scientific), respectively. Cells were stained with 20 μM DCFDA or 5 μM MitoSOX™ Red for 30 min at 37 °C. Thereafter, cells were washed and collected in PBS/5%FBS for analysis. Ten thousand gated events were analyzed by flow cytometry on a CytoFlex S (Beckman Coulter, Brea, CA, USA), using the FITC channel for DCFDA and PE channel for MitoSOX™ Red. The median fluorescence intensity of cells was determined using CytExpert 2.0 software (Beckman Coulter).

### 2.11. Autophagy Assay

Autophagy levels were assessed using CYTO-ID^®^ Autophagy detection kit (Abcam). Cells were washed and the medium was replaced with the assay buffer. Samples were stained with 1:1000 CYTO-ID^®^ probe and placed at 37 °C for 30 min. Cells were collected, washed, and fixed in 10% formaldehyde solution for 20 min at room temperature. Samples were washed again and collected in assay buffer for analysis. Ten thousand gated events were analyzed by flow cytometry on a CytoFlex S (Beckman Coulter, Brea, CA, USA), using the FITC channel. The median fluorescence intensity of cells was determined using CytExpert 2.0 software (Beckman Coulter).

### 2.12. Lipid Peroxidation Measurement

Lipid peroxidation analysis was assessed by the Lipid Peroxidation (MDA) Assay kit (Abcam) according to the manufacturer’s instructions. Briefly, 2 million cells were collected and resuspended in 250 μL of MDA Lysis Buffer +BHT and homogenized by sonication. Samples were centrifuged at 13,000× *g* for 10 min at 4 °C. Then, 200 ul of supernatant were added to 600 µL of TBA solution and placed at 95 °C for 1 h. After cooling down, fluorescence was measured at excitation/emission wavelengths of 532 nm and 553 nm, respectively, using a Cary Eclipse Fluorescence Spectrophotometer (Agilent Technologies).

### 2.13. Differential Correlation Analysis (DCA)

The differential correlation (DCA) analysis [22] is an emerging approach used to gain insights into the significant difference in correlations between pairs of molecular identifiers (e.g., genes, proteins, metabolites) across multiple conditions (e.g., disease and non-diseased condition or treated versus non-treated condition). Notably, DCA operates on the level of entity pairs rather than individual entities and allows for revealing dependencies by identifying coordinated expressions that differ across two conditions of interest. In the present study, DCA was exploited to investigate differential correlations between the control condition (CTR) and the erastin-treated condition (ERASTIN) and it was performed through the DCGA R package 1.0.3 (R version 4.1.3) [23].

### 2.14. RNA Extraction and Real-Time PCR

Total RNA was isolated using TRIzol reagent (Life Technologies) and it was reverse transcribed to cDNA using a High-Capacity cDNA Reverse Transcription Kit using a commercial TranScriba Kit (A&A Biotechnology, Gdańsk, Poland), following the manufacturer’s instructions. The real-time PCRs were performed in triplicate for each data point using the CFX Connect (Bio-Rad Laboratories, Milan, Italy) and Sybr Green technique. The oligonucleotides used are shown in Table S1. The changes in target mRNA content in relation to the β-actin housekeeping gene were determined using the ΔΔct Method for all cell lines. The data are presented as the mean values ± standard deviation (SD) of three independent experiments and were statistically analyzed using a *t*-test or one- or two-way analysis of variance (ANOVA), followed by Dunnett’s or Bonferroni’s multiple comparisons.

### 2.15. cDNA Microarray Expression Analysis

Total RNA for microarray analysis was extracted using the RNeasy mini kit (Qiagen, Hilden, Germany), and its concentration, purity, and RNA integrity, measured as RNA integrity number (RIN), were assessed using a TapeStation 4150 (Agilent Technologies). Then, 100 ng of total RNA were applied for Cy3-labeling reaction using the one-color Quick Amp Labeling protocol (Agilent Technologies). Labeled cRNA was hybridized to a SurePrint G3 Human Gene Expression v3 8x60K Microarray Gene Chip (Agilent Technologies) for 16 h at 68 °C and scanned using a SureScan Microarray Scanner (Agilent Technologies). Expression values were calculated by the software package Feature Extraction 12.2.0.7 (Agilent Technologies). Statistical analysis of the expression data was performed using the Gene Spring GX 13.0 Software (Agilent Technologies). For the analysis, raw intensities were log2 transformed and the Mann–Whitney unpaired test with an adjusted FDR *p*-value < 0.01 was applied. Genes expression heatmaps for specific pathways were obtained, selecting the genes of interest using the Reactome Pathways [24] list of genes for each pathway of interest as a reference. Heatmaps were generated using the Seaborn graphical library [25]. The lists of genes used for the heatmaps are provided in File S1. Transcriptional data presented in this work have been deposited in the National Center for Biotechnology Information Gene Expression Omnibus (GEO) (https://www.ncbi.nlm.nih.gov/geo/, accessed on 25 March 2025) and are accessible through GEO Series accession numbers GSE292890 and GSE292891.

### 2.16. Integration Analysis Between Transcriptomics and Metabolomics Data

The integration between transcriptomics and metabolomics data were obtained through the WEB-based GEne SeT AnaLysis Toolkit (https://www.webgestalt.org/, accessed on 4 December 2024) [26]. Over-representation analysis was performed using the statistically significant lists of genes and metabolites obtained from the two different analyses using “Reactome pathways” as functional databases. Parameters for the enrichment analysis were as follows: Minimum number of IDs in the category. 5; maximum number of IDs in the category: 2000; FDR method: Benjamini–Hochberg; significance level: FDR < 0.05.

## 3. Results

### 3.1. Pancreatic Cancer Cell Lines Exhibit Great Metabolic Heterogeneity

Pancreatic ductal adenocarcinoma (PDAC) cell lines display remarkable metabolic heterogeneity, reflecting diverse adaptive strategies under adverse conditions. To investigate how metabolic rewiring supports cellular adaptation and contributes to variability in drug response, we perform a detailed metabolic analysis of eight PDAC cell lines selected to represent a broad spectrum of intrinsic metabolic phenotypes (Figure S1A). This panel reflects the metabolic heterogeneity characteristic of human pancreatic tumors.

**Figure 1:**
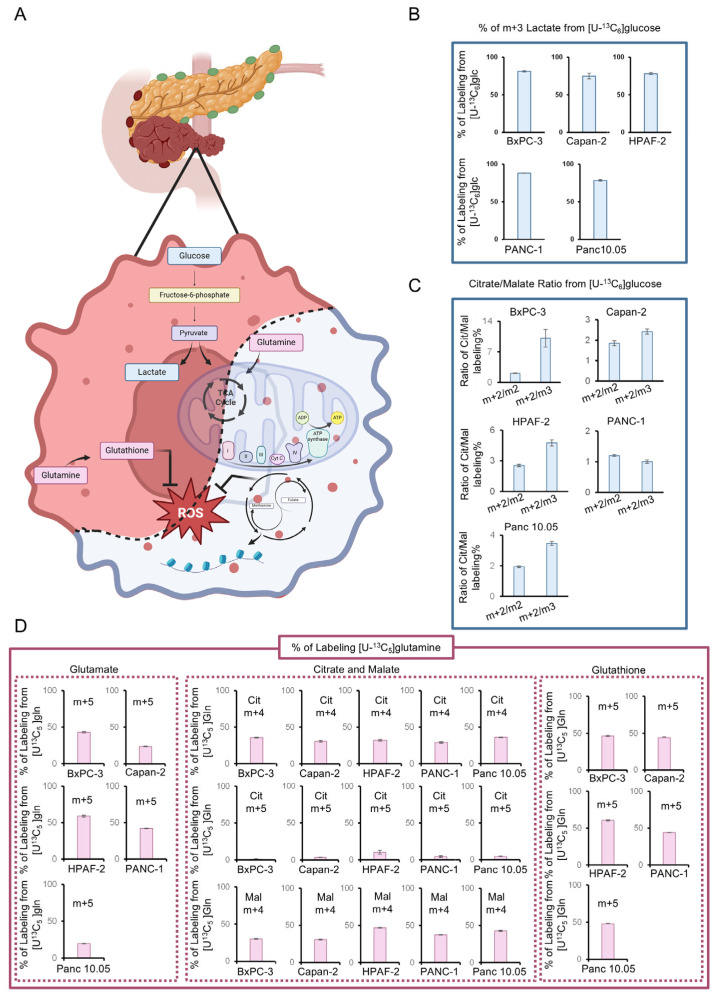
Metabolic characterization of PDAC cell lines. (**A**) Schematic representation of the main pathways involved in metabolic rewiring in PDAC cell lines. The red part of the cell indicates the conserved profile of glucose-derived metabolites across all PDAC cell lines, while the blue part of the cell indicates the variability in mitochondrial function. Created in https://BioRender.com. (**B**) Percentage of m+3 lactate derived from [U-^13^C_6_]glucose in BxPC-3, Capan-2, HPAF-2, PANC-1, and Panc 10.05 obtained by LC-MS analysis. (**C**) m+2 Citrate/m+2 Malate and m+2 Citrate/m+3 Malate ratios derived from [U-^13^C_6_]glucose in BxPC-3, Capan-2, HPAF-2, PANC-1, and Panc 10.05 obtained by LC-MS analysis. (**D**) Percentage of m+5 glutamate, m+4 citrate, m+4 malate, and m+5 glutathione derived from [U-^13^C_5_]glutamine in BxPC-3, Capan-2, HPAF-2, PANC-1, and Panc 10.05 obtained by LC-MS analysis. All data are expressed as means ± SD.

Basic stratification of PDAC cell lines, assessed through long-term proliferation curves under nutrient deprivation (glucose or glutamine starvation), confirms universal glucose dependency across all cell lines, while only PANC-1 exhibits strong glutamine dependency (Figure S1A). Among the tested cell lines, PANC-1, HPAF-2, Panc 10.05, and Capan-2 demonstrate the highest proliferation rates, underscoring metabolic variability in growth dynamics. This adaptability aligns with the exceptional metabolic heterogeneity revealed through untargeted profiling (Figure S1B). Metabolic signature analysis indicates that while some metabolites follow similar trends, others exhibit pronounced differences, suggesting distinct metabolic adaptations among PDAC cell lines (Figure S1B). Notably, metabolites involved in nucleotide and amino acid metabolism show consistent patterns, highlighting a shared reliance on redox homeostasis and anaplerotic reactions. Additionally, a conserved profile of glucose-derived metabolites across all PDAC cell lines confirms a predominant glycolytic metabolism, despite variability in mitochondrial function (Figure 1A and Figure S1B–D). In contrast, fluctuations in key metabolites such as NADH, Coenzyme A, Riboflavin, and ATP underscore differences in mitochondrial function and oxidative phosphorylation reliance. Moreover, variability in one-carbon and nucleotide metabolism (Folate, S-Adenosylmethioninamine, SAICAR), as well as shifts in Kynurenine and Erythrose 4-phosphate levels, may indicate metabolic alterations related to immune modulation and pentose phosphate pathway activity (Figure S1B). Stable isotope labeling in metabolically heterogeneous PDAC cell lines (BxPC3, Capan-2, HPAF-2, PANC-1, and Panc 10.05) further validates the metabolic flexibility of PDAC cells. Across all analyzed cell lines, using a [U-^13^C_6_]glucose tracer, we observe a conserved glucose oxidation pathway via lactate, but with substantial variability in mitochondrial metabolism (Figure 1A–D). Moreover, monitoring TCA cycle flux, we observe an increased citrate m+2/malate m+3 labeling ratio in most of PDAC cell lines analyzed, except for Panc-1 (Figure 1C). The extent to which malate is derived from the non-canonical TCA cycle can be represented by this ratio, as m+2 citrate originates from pyruvate dehydrogenase (PDH) activity, while m+3 malate results from pyruvate carboxylase (PC). The observed increase suggests that glucose metabolism contributes to metabolic heterogeneity by altering TCA cycle routing (Figure 1C). Meanwhile, although only PANC-1 exhibits glutamine addiction (Figure S1A), the significant variability in glutamate m+5 labeling from [U-^13^C_5_]glutamine suggests differential glutamine metabolism across PDAC cell lines. Forward TCA cycle activity is evident in all PDAC cells analyzed, with similar labeling patterns observed in glutathione (GSH) (Figure 1A,D). Collectively, these findings highlight the metabolic flexibility of PDAC cells, enabling diverse nutrient utilization strategies to support survival and growth.

Together, these findings underscore the metabolic plasticity of PDAC cells, allowing diverse nutrient utilization strategies that support growth and complicate their classification into distinct metabolic subgroups [27]. This metabolic flexibility may contribute to therapy resistance, emphasizing the need to better understand metabolic pathway alterations and their role in adaptive responses.

### 3.2. Pancreatic Cancer Metabolism Shows Sensitivity to Erastin, but It Is Not Enough

**Figure 2:**
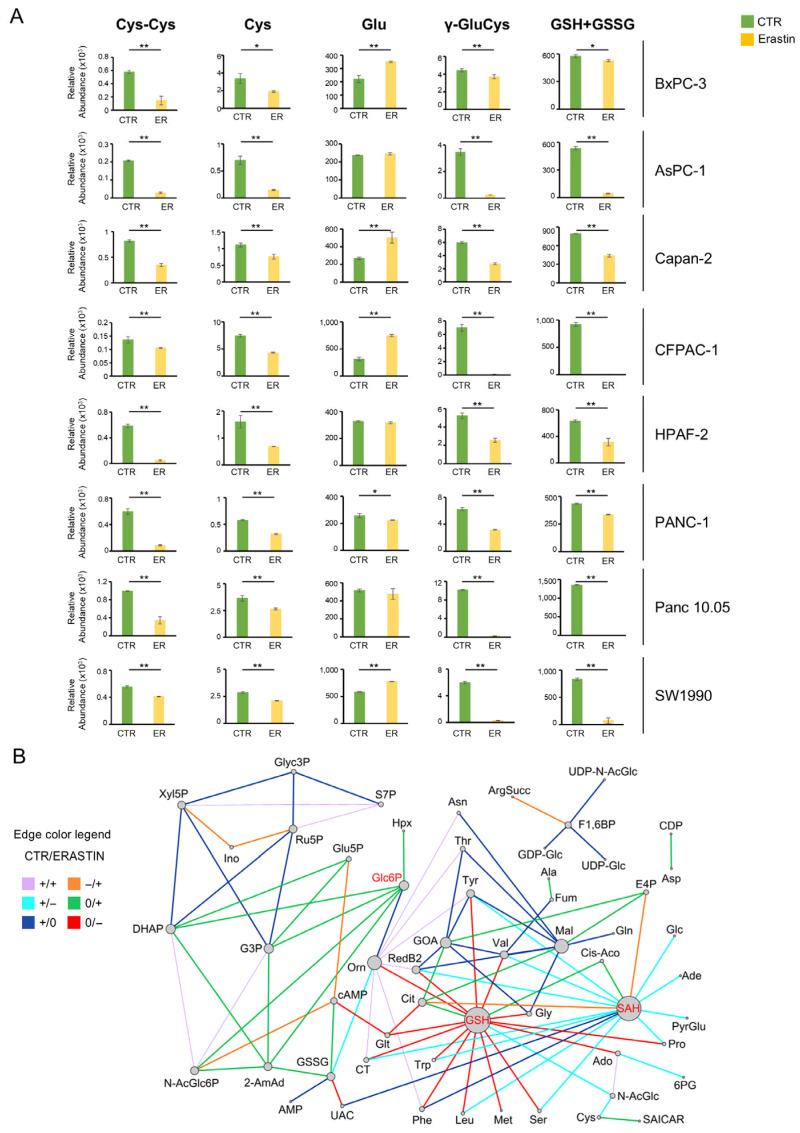
Metabolic characterization of PDAC cell lines after erastin treatment. (**A**) Relative abundance of metabolites involved in glutathione synthesis (cystine, cysteine, glutamate, gamma-glutamyl cysteine, and reduced and oxidized glutathione) obtained by LC-MS in the eight indicated PDAC cell lines under erastin treatment (n = 3) * *p* ≤ 0.05, ** *p* ≤ 0.01. (**B**) Differential correlation analysis (DCA). Network of the top 100 differential correlations (edges) computed among metabolites (nodes) moving from the control condition (CTR) to the erastin-treated condition (ERASTIN). The differential correlations were considered statistically significant if the *p*-value was less than 0.05 and they were ranked according to the *p*-values. The size of network nodes is proportional to their degree. The color of the network edges represents the different classes used by DCA and reported in legend where + means a significant (*p*-value < 0.05) and positive correlation; − means a significant (*p*-value < 0.05) and negative correlation; 0 means a non-significant correlation (*p*-value >= 0.05). Classes without a switch between CTR and ERASTIN (i.e., +/+ and −/−) are represented with less thick edges. Metabolites of interest are highlighted in red.

Given the metabolic flexibility of PDAC cells, we investigated the effects of erastin treatment (see scheme of action in Figure S2A and Table S2) to better understand the distinct metabolic adaptations that influence the efficacy of this treatment and its associated adaptive mechanism. The selected panel of PDAC cell lines reflects the patient tumor landscape, with seven out of eight lines harboring KRAS mutations, while BxPC-3 is the only RAS wild-type cell line. To assess erastin sensitivity, we first determined the IC_50_ values for all eight PDAC cell lines after 48 h of treatment (see Figure S2B). As shown in Figure S2B, IC_50_ values ranged from 0.6 µM to 8 µM, with the RAS wild-type BxPC-3 exhibiting the highest IC_50_, indicating lower sensitivity to erastin. As a direct readout, analysis of lipid peroxidation using the specific IC_50_ concentration for each cell line confirms significant ferroptosis activation in all PDAC cell lines, as indicated by increased levels of malondialdehyde (MDA) (Figure S2C). Consistent with action of erastin mechanism, which disrupts metabolic pathways involved in GSH synthesis, a significant decrease in the levels of metabolites involved in GSH biosynthesis is observed in all K-Ras-mutated PDAC cells, compared to K-Ras wild type BxPC-3 (Figure 2A), while divergent treatment responses are observed for the intracellular glutamate levels (Figure 2A).

According to the great heterogeneity reported in Figure S1C,D, the mitochondrial oxygen consumption rate (OCR) shows a decreased level of basal respiration under erastin treatment in CFPAC-1, BxPC-3, Panc 10.05, and Capan-2, whereas the same parameter is not affected in the case of AsPC-1, HPAF-2, SW1990, and PANC-1 (Figure S3). Interestingly, besides basal respiration, mitochondrial ATP production, maximal respiration, and respiratory spare capacity are dramatically reduced in CFPAC-1 and Capan-2 cell lines treated with erastin (Figure S3). Although the long-term cell proliferation curves demonstrate a substantial decrease in cellular growth following 48 h treatment, a recovery of growth is observed at later time points (see Figure S4). While the described results confirm the effectiveness of erastin on PDAC cells, a single treatment is insufficient to completely inhibit their proliferation and disrupt mitochondrial respiration. Therefore, to better uncover metabolic dependencies across different PDAC cells and rationalize metabolic modulators involved under erastin treatment, we applied the differential correlation analysis (DCA) computational tool (Figure 2B). DCA was performed using relative metabolites abundance levels of all PDAC cells grown in the absence and in the presence of erastin for 48 h. The analysis identifies a network of the top 100 differential correlations (edges) computed among metabolites (nodes) moving from the control condition (CTR) to the erastin-treated condition. Noteworthy, DCA identifies glucose 6-phosphate -G6P-, glutathione -GSH-, and S-adenosylhomocysteine -SAH- as main metabolic hubs (Figure 2B). While the identification of GSH as a metabolic hub in erastin treatment was largely anticipated, the discovery of G6P and SAH as strategic points represents a more intriguing finding. In particular, G6P shows a strong direct positive correlation during erastin treatments with glycolysis (G3P and DHAP), HBP (N-Ac-Glc6P), nucleotides metabolism (Hpx and Orn), and signal transduction by cAMP, compared to CTR (Figure 2B). Differently from G6P, SAH shows a direct negative correlation with amino acids, redox, and nucleotide metabolism during erastin treatment (Figure 2B). These interactions, identifying detailed metabolic variation features under erastin treatment, could explain the failure of single-drug treatment observed at late time points. Consequently, these findings offer significant information to guide the selection of additional metabolic drugs to be tested in combination with erastin.

### 3.3. What Doesn’t Kill Makes Stronger: Metabolic Flexibility Biomarkers to Select Combinatorial Metabolic Drug-Targets

**Figure 3:**
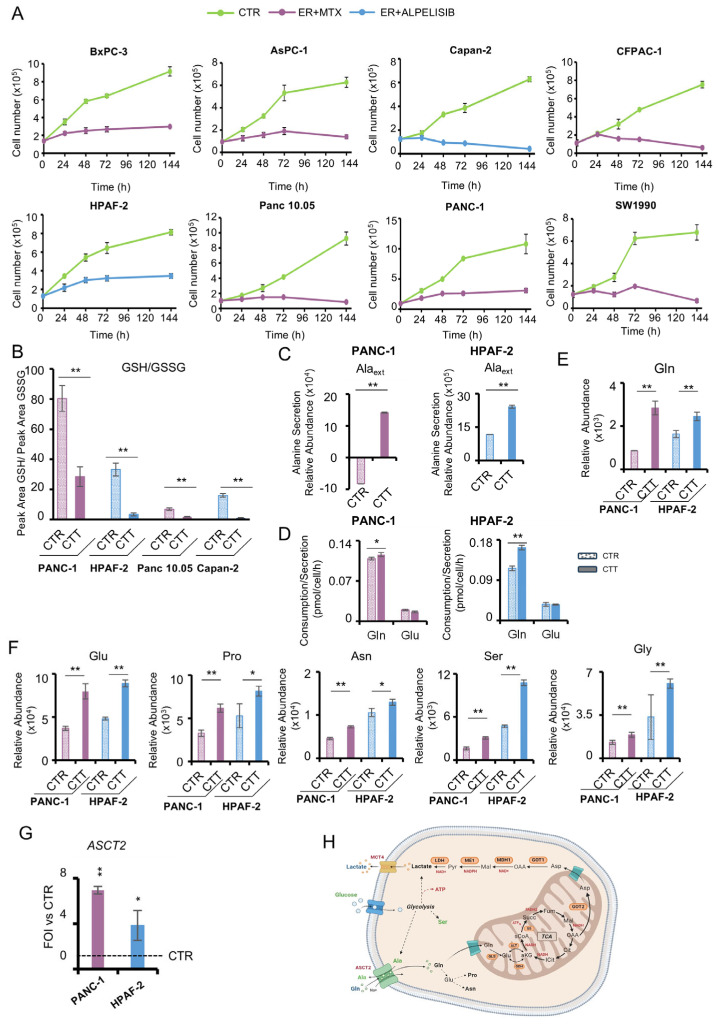
Metabolic characterization of PDAC cell lines after combinatorial treatment. (**A**) Proliferation curves of eight PDAC cancer cell lines in normal growth condition (**―**), in the presence of both erastin and methotrexate (**―**), or in the presence of both erastin and alpelisib (**―**). Cells were collected and counted at the indicated time points. (**B**) GSH/GSSG ratio measured by dividing the peak area of the GSH signal by the peak area of the GSSG signal in PANC-1, HPAF-2, Panc 10.05, and Capan-2 in normal growth condition or with combinatorial treatments obtained by LC-MS. (**C**) Alanine secretion in the extracellular medium of PANC-1 (**left panel**) and HPAF-2 (**right panel**) in the presence or absence of combinatorial treatment obtained by GC-MS. (**D**) Extracellular glutamine uptake and glutamate secretion determined enzymatically using a YSI2950 bioanalyzer in PANC-1 (**left panel**) and HPAF-2 (**right panel**) in the presence or absence of combinatorial treatment. (**E**) Relative glutamine abundance in PANC-1 (**left panel**) and HPAF-2 (**right panel**) in normal growth conditions or with combinatorial treatment obtained by LC-MS. (**F**) Relative glutamate, proline, asparagine, serine, and glycine abundance in PANC-1 and HPAF-2 in normal growth conditions or with combinatorial treatment obtained by LC-MS. (**G**) *ASCT2* gene expression assessed by real-time qPCR in PANC-1 and HPAF-2 cell lines after combinatorial treatment. (**H**) Schematic representation of glucose and glutamine diversion in PDAC. Plasma membrane transporters ASCT2 and MCT4 are represented as glutamine/alanine exchanger and lactate exporter, respectively. Dotted lines are representative of multistep pathways. ATP and reducing equivalent molecules produced by glutamine and glucose diversion are indicated in red. In green are Ala and Ser deriving from glucose metabolism. Created in https://BioRender.com. In all the panels, ■ stands for erastin+methotrexate and ■ for erastin+alpelisib. All data are expressed as means ± SD. * *p* ≤ 0.05, ** *p* ≤ 0.01.

In order to enhance the efficacy of erastin treatment and in line with the identification of G6P and SAH as metabolic hubs, four distinct inhibitors were selected to specifically target the metabolic pathways identified through DCA (Figure 2B and Figure S5). These inhibitors were employed in combination with erastin on the panel of PDAC cells. In particular, we specifically selected the following: (i) alpelisib, a PI3K inhibitor, involved in nutrients uptake, metabolism, proliferation, and survival [28]; (ii) everolimus, an inhibitor of mTOR serine/threonine kinase that acts as a glycolysis inhibitor [29]; (iii) resveratrol, a polyphenolic compound, FDA approved, able to inhibit glucose uptake, glycolysis via lactate, and enhances pyruvate dehydrogenase activity [30]; and (iv) methotrexate, an antimetabolite able to inhibits one carbon and nucleotides metabolism [31]. The selection of the first three inhibitors was based on the identification of G6P as a metabolic hub, while the selection of methotrexate was influenced by the identification of SAH, which is involved in one-carbon metabolism. To identify the best drug to use in combination with erastin, the IC_50_ of the selected inhibitors for each PDAC cell line was first measured after 48 h of treatment (Figure S5). Overall, the collected data identify methotrexate and alpelisib as the inhibitors with the best efficiency–concentration ratio (Figure S5). We selected methotrexate for PANC-1, Panc 10.05, SW1990, CFPAC-1, BxPC-3, and AsPC-1, while alpelisib for HPAF-2 and Capan-2 were used in combination with erastin. Long-term proliferation curves confirm that erastin is much more efficient when associated with methotrexate or alpelisib at late time points (Figure 3A).

PANC-1, Panc 10.05, HPAF-2, and Capan-2 cell lines were selected as representatives of the PDAC panel to investigate the metabolic flexibility under the two combinatorial treatments (CTT), erastin–methotrexate and erastin–alpelisib (Figure 3). Indeed, these cell lines are characterized by the highest proliferation rate (Figure S1A), the highest degree of variability in response to single erastin treatment (Figures S3 and S4), and different sensitivity to combinatorial drugs, being PANC-1- and Panc 10.05-responsive to methotrexate and HPAF-2- and Capan-2-responsive to alpelisib (Figure 3A).

The significant reduction in the GSH/GSSG ratio (Figure 3B) indicates that redox metabolism remains markedly compromised even in the presence of the CTT, thus preserving the effect observed in the presence of erastin alone. While the extraction protocol was not specifically optimized for redox stabilization, the consistent sample processing across all conditions supports the validity of the observed relative differences in glutathione redox status, which were calculated based on the peak areas of GSH and GSSG signals.

To further evaluate the effect of combinatorial treatments, metabolic analyses were conducted at both intracellular and extracellular levels. In the media analysis, the CTT samples exhibited a markedly elevated extracellular alanine abundance (Figure 3C and Figure S6A) concomitant with enhanced extracellular glutamine consumption (Figure 3D and Figure S6B) compared to the control conditions. This finding is in good agreement with the higher level of glutamine observed at the intracellular level (Figure 3E and Figure S6C). Moreover, increased levels of non-essential amino acids (NEEA) Glu, Pro, Asn, Ser, and Gly are observed in CTT samples (Figure 3F and Figure S6D). These metabolites are involved in anabolic processes, ROS scavenger pathways, and ammonia detoxification. At the molecular level, we also observe an over-expression of the plasma membrane transporter ASCT2 (*SLC1A5*) (Figure 3G and Figure S6E). This transporter is one of the key players in the accumulation of intracellular glutamine in cancer cells, with a mechanism that allows entry of a five carbon atoms molecule, i.e., glutamine, in exchange with smaller substrates (three carbon atoms molecules), such as alanine [32] (Figure 3H), consolidating a glutamine diversion hypothesis and further highlighting the metabolic flexibility of PDAC cells under stress conditions.

### 3.4. Combinatorial Drug Treatment Induces a New Metabolic Rewiring in Pancreatic Cancer Cells That Involves Serine Metabolism

**Figure 4:**
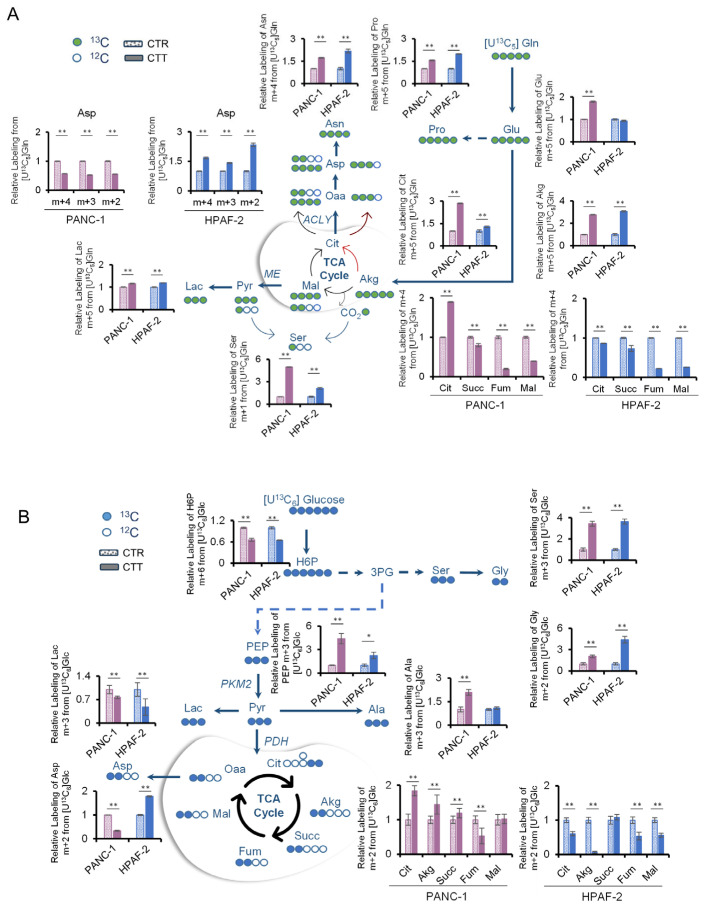
Metabolic characterization of PDAC cell lines after combinatorial treatment by stable-isotope tracing analyses. (**A**,**B**) Schematic representations and atomic transition map of relative isotope labeling enrichment of metabolites from [U-^13^C_6_]glucose (**blue circles**) (**A**) and [U-^13^C_5_]glutamine (**green circles**) (**B**) in PANC-1 and HPAF-2 in the presence of combinatorial treatment (■ for erastin+methotrexate; ■ for erastin+alpelisib) obtained by LC-MS analysis. Filled circles indicate 13C enrichment. Data are expressed as relative to control. (n = 3). All data are expressed as means ± SD. * *p* ≤ 0.05, ** *p* ≤ 0.01.

To better evaluate the role of glutamine in the metabolic rewiring after the combinatorial treatment, metabolic labeling analysis was performed using [U-^13^C_5_]glutamine of PANC-1 and HPAF-2, chosen as prototypes of the two different CTT, due to their higher proliferation rate (Figure 3A) under erastin–methotrexate and erastin–alpelisib, respectively (Figure 4A). Consistently with the above-described results (Figure 3F and Figure S6D), the labeling analyses showed an increased synthesis from glutamine of the NEAA glutamate, proline, and asparagine in CTT. Furthermore, it is important to note the increased m+3 lactate in treated cells in comparison to the control group, which is indicative of enhanced glutaminolysis. A further peculiarity is the increased level of m+5 citrate, which suggests that the glutamine-dependent reductive carboxylation pathway is more active in CTT cells. A surprising novelty was observed in the m+1 serine labeling from [U-^13^C_5_]glutamine, which was significantly elevated under CTT in both cancer cell lines. Indeed, serine can be synthesized de novo from 3-phosphoglycerate, which in turn can be obtained from the gluconeogenic route by recycling ^13^CO_2_ from the decarboxylation of [U-^13^C_5_]glutamine as demonstrated by [33] (Figure 4A). Given this unexpected result, a more detailed investigation into the role of serine in these treatments was undertaken. The study aimed to explore whether its de novo synthesis was also increased through the glycolytic pathway. Labeling experiments using [U-^13^C_6_]glucose were performed. These experiments revealed higher levels of m+3 serine in the treated cells, as well as increased levels of m+2 glycine, derived from serine (Figure 4B). In addition to the effects on serine synthesis, further diversion of canonical glucose oxidation (via lactate) toward anabolic processes was observed. Indeed, significantly decreased levels of m+3 lactate and increased levels of m+3 serine and m+3 alanine were measured in PANC-1 under CTT (Figure 4B). The observed behavior was similar for HPAF-2, except for m+3 alanine levels, which remain constant between the control and CTT samples (Figure 4B). Besides these commonalities, the two cancer cell lines showed some intriguing differences that further highlight the profound metabolic rewiring that characterizes PDAC. PANC-1 and HPAF-2 have different metabolic behaviors regarding TCA cycle labeling (Figure 4B). A contrasting pattern was also evident in m+2 aspartate labeling, decreasing in PANC-1-treated cells and increasing in HPAF-2 (Figure 4B). The same occurs with m+4, m+3, and m+2 aspartate using [U-^13^C_5_]glutamine (Figure 4A).

**Figure 5:**
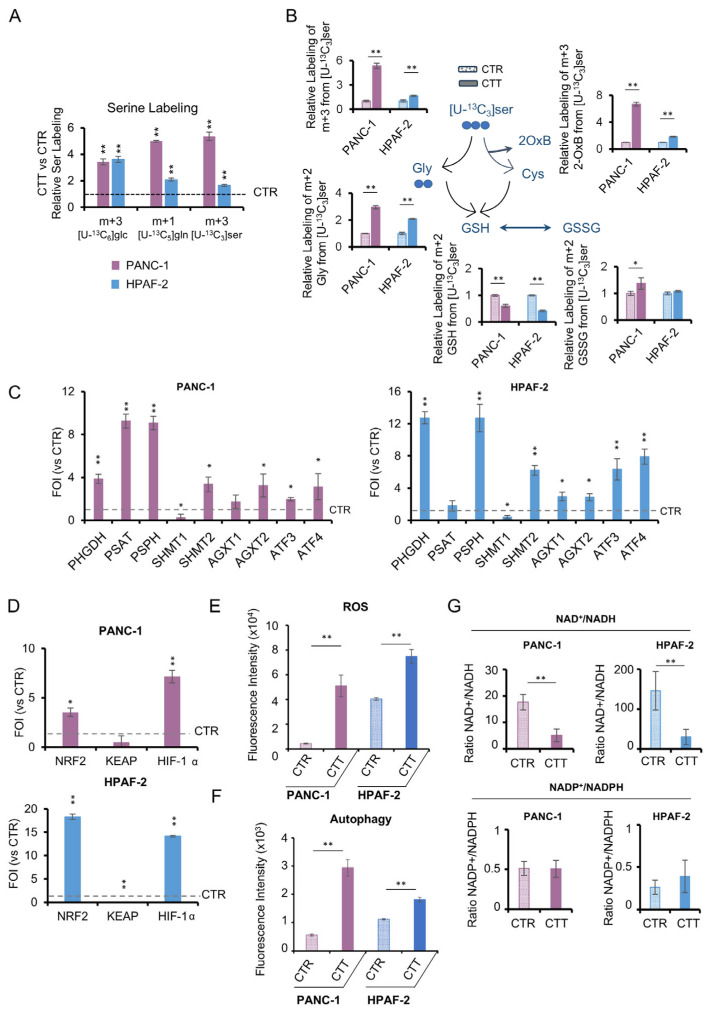
Serine synthesis pathway and redox metabolism rewiring in PDAC cell lines after combinatorial treatment. (**A**) Relative serine labeling from [U-^13^C_6_]glucose, [U-^13^C_5_]glutamine, and [U-^13^C_3_]serine in PANC-1 and HPAF-2 in the presence of combinatorial treatment obtained by LC-MS analysis. Data are expressed as relative to control (n = 3). (**B**) Schematic representations and atomic transition map of relative isotope labeling enrichment of metabolites of cysteine and glutathione synthesis from [U-^13^C_3_]serine in PANC-1 and HPAF-2 in the presence of combinatorial treatment obtained by LC-MS analysis. Filled circles indicate 13C enrichment. Data are expressed as relative to control. (n = 3). (**C**) Expression of genes related to de novo serine synthesis pathway assessed by real-time qPCR in PANC-1 and HPAF-2 cell lines after combinatorial treatment. The molecular data were normalized to β-actin, and the ΔΔct values were expressed as the fold of induction (FOI) of the ratio between treated and control cells. (**D**) Expression of Nrf2, KEAP, and HIF-1α assessed by real-time qPCR in PANC-1 and HPAF-2 cell lines after combinatorial treatment. The molecular data were normalized to β-actin, and the ΔΔct values were expressed as the fold of induction (FOI) of the ratio between treated and control cells. (**E**) Total ROS levels measured by DCFDA staining in PANC-1 and HPAF-2 in normal growth condition or in the presence of combinatorial treatment. (**F**) Autophagy levels assessed by the CYTO-ID^®^ Autophagy detection kit in PANC-1 and HPAF-2 in normal growth condition or in the presence of combinatorial treatment. (**G**) NAD^+^/NADH ratio (**upper panel**) and NADP^+^/NADPH ratio (**lower panel**) in PANC-1and HPAF-2 in normal growth conditions or in the presence of combinatorial treatment obtained by LC-MS (n = 3). All data are expressed as means ± SD. * *p* ≤ 0.05, ** *p* ≤ 0.01. In all the panels, ■ stands for erastin+methotrexate and ■ for erastin+alpelisib. All data are expressed as means ± SD. * *p* ≤ 0.05, ** *p* ≤ 0.01.

In addition to being produced through de novo synthesis, serine is a non-essential amino acid that can be imported from the medium. To investigate whether increased synthesis is paired with increased uptake, experiments were conducted in the presence of the [U-^13^C_3_]serine tracer (Figure 5A). Even in this case, CTT samples showed higher m+3 serine levels than control samples. As further proof, we found significantly increased m+2 Gly and m+3 2-oxobutanoate product of cysteine synthesis from labeled [U-^13^C_3_]serine (via cystathionine), used to maintain the redox balance through glutathione and NADPH synthesis (Figure 5B). Taken together, these data demonstrate the activation of serine metabolism with combinatorial drug treatments, suggesting a key role for serine as a metabolic CTT stress biomarker.

### 3.5. The Activation of De Novo Serine Synthesis Pathway (SSP) Reveals Pancreatic Cancer Resilience to Combinatorial Drug Treatments

In order to verify the hypothesis that the combinatorial treatments can activate serine synthesis as part of an adaptive metabolic response, we assessed the expression profile of the de novo SSP genes in four PDAC cell lines. These cell lines (Panc-1, HPAF-2, Panc 10.05, and Capan-2) were selected from the original panel of eight based on their high proliferation rates, metabolic adaptability, and differential sensitivity to methotrezate and/or alpelisib. Interestingly, the results reveal a pronounced activation of SSP genes (Figure 5C and Figure S6F), along with a parallel activation of genes associated with anabolic processes and redox metabolism for NADPH generation (Figure S6G). The same trend is also evident in the significantly increased expression of ATF3 and ATF4, essential for transcriptional activation of SSP genes (Figure 5C and Figure S6F). Notoriously, these factors are activated under various cellular stresses, including serine deprivation [34].

Treated cells further exhibit ROS levels (Figure 5E, upper panel, and Figure S6I), which coincide with over-expression of Nrf2 and HIF1α and downregulation of their negative regulator KEAP downregulation (Figure 5D and Figure S6H). It is noteworthy that this gene remodeling is observed in three out of the four lines, with the exception of Panc 10.05, in which the activation pattern present in the other lines is not evident. In parallel, treated cells show increased levels of autophagy (Figure 5F and Figure S6J) and an imbalance in the NAD^+^/NADH ratio (Figure 5G, upper panel). In the opposite way to NAD^+^/NADH ratio levels, PDAC cells show a perfect balance of NADP+/NADPH ratio between CTT and CTR (Figure 5G, lower panel). Consistently with our published data, in which NADPH crashing strongly reduced tumor growth in vitro and in vivo [35], these results sustain the hypothesis of metabolic adaptation of PDAC cells through de novo SSP activation and uptake to maintain redox homeostasis [36]. In this scenario, the increased SSP activation, linked to abnormal cellular nucleotide and lipid metabolism, mitochondrial function, and epigenetic modifications, may drive drug resistance in PDAC cells.

### 3.6. Metabolic and Transcriptional Rewiring Drives Adaptive Resilience to a Second Round of Treatment in PDAC Cells

**Figure 6:**
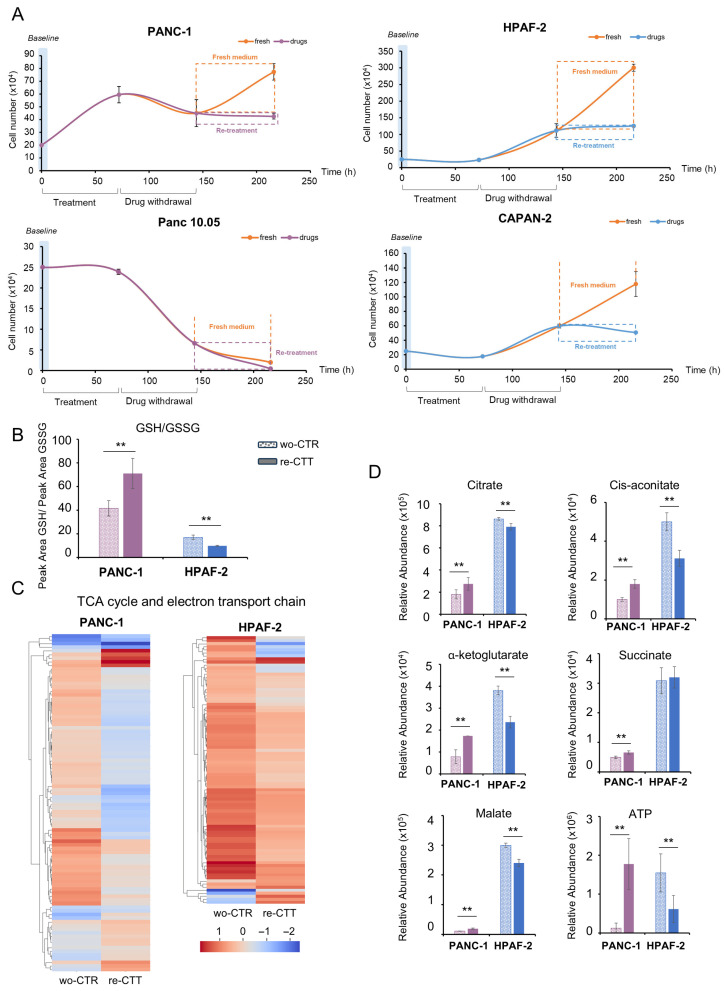
Transcriptional and metabolic rewiring in PDAC cell lines after a second round of treatment. (**A**) Proliferation curves of PANC-1, HPAF-2, Panc 10.05, and Capan-2 cell lines. Cells were exposed to combinatorial treatment for 72 h, followed by 72 h of drug withdrawal. Cells were then cultured in normal growth conditions or in the presence of combinatorial treatment. Cells were collected and counted at the indicated time points. (**B**) The GSH/GSSG ratio was measured by dividing the peak area of the GSH signal by the peak area of the GSSG signal in PANC-1, and HPAF-2 in wash-out control or in re-treatment condition obtained by LC-MS. (**C**) Hierarchical clustering of statistically significant genes involved in the TCA cycle and electron transport chain pathway in PANC-1 and HPAF-2 in the wash-out control condition or in re-treatment condition obtained by microarray analysis. (**D**) Relative abundance of the TCA cycle intermediates in PANC-1 and HPAF-2 in the wash-out control condition or in re-treatment condition obtained by LC-MS. In all the panels, ■ stands for erastin+methotrexate and ■ for erastin+alpelisib. All data are expressed as means ± SD. ** *p* ≤ 0.01.

To further investigate the persistance of adaptive phenotype following drug withdrawal, we analyzed PDAC cells after the removal of both drugs from the culture medium (Figure 6A).

Four representative PDAC cell lines were initially treated with CTT for 72 h, followed by a 72 h washout with fresh media. After this washout period, the cells were either re-treated with CTT for an additional 72 h (re-CTT) or left untreated (wo-CTR). Proliferation curves indicate that only the cell lines exhibiting SSP activation (PANC-1, Capan-2, and HPAF-2) demonstrate the capacity to restore growth after 72 h of treatment. In contrast, Panc 10.05 displays an ineffective recovery. Although proliferation is rescued, these cell lines remain susceptible to a second round of treatment, but with less impairment in proliferative capacity compared to the first round (Figure 6A). To gain deeper insights into this phenomenon, we conducted more in-depth analyses on two representative cell lines, PANC-1 and HPAF-2, under the two latest conditions: washed-out cells (wo-CTR) and cells subjected to a second round of treatment (re-CTT). Consistent with previous findings, re-CTT HPAF-2 cells exhibit a significant reduction in the GSH/GSSG ratio compared to wo-CTR cells, indicating increased oxidative stress (Figure 6B). Unexpectedly, PANC-1 re-CTT cells show an elevated GSH/GSSG ratio, suggesting a more effective antioxidant response or reduced drug efficacy in disrupting redox homeostasis (Figure 6B). Moreover, the unchanged NAD+/NADH ratio in both cell lines following re-treatment (Figure S6K) suggests a compensatory mechanism that maintains redox balance despite drug exposure, potentially contributing to reduced re-treatment sensitivity.

To further elucidate the impact of the second round of treatments, we conducted a comprehensive analysis integrating transcriptional and metabolomic data. Differentially expressed genes and statistically significant metabolites between the re-CTT and wo-CTR conditions are used for a multi-omics integration analysis. The results of the integrated over-representation analysis are presented in Figure S7A and Figure S7B, respectively. In addition to the generic enriched pathways common to both lines, which are related to gene expression, translation, signal transduction, cell cycle, and protein metabolism, several more strictly metabolic pathways are highlighted. For instance, the TCA cycle and respiratory electron transport pathway are identified in both cell lines by the enrichment analysis, yet the effects of these pathways differ in the two cell lines (Figure 6C,D). In addition to these pathways, several other mechanisms are unique to each cell line.

#### 3.6.1. PANC-1: Metabolic Flexibility and Survival Signaling Under Drug Re-Treatment

**Figure 7:**
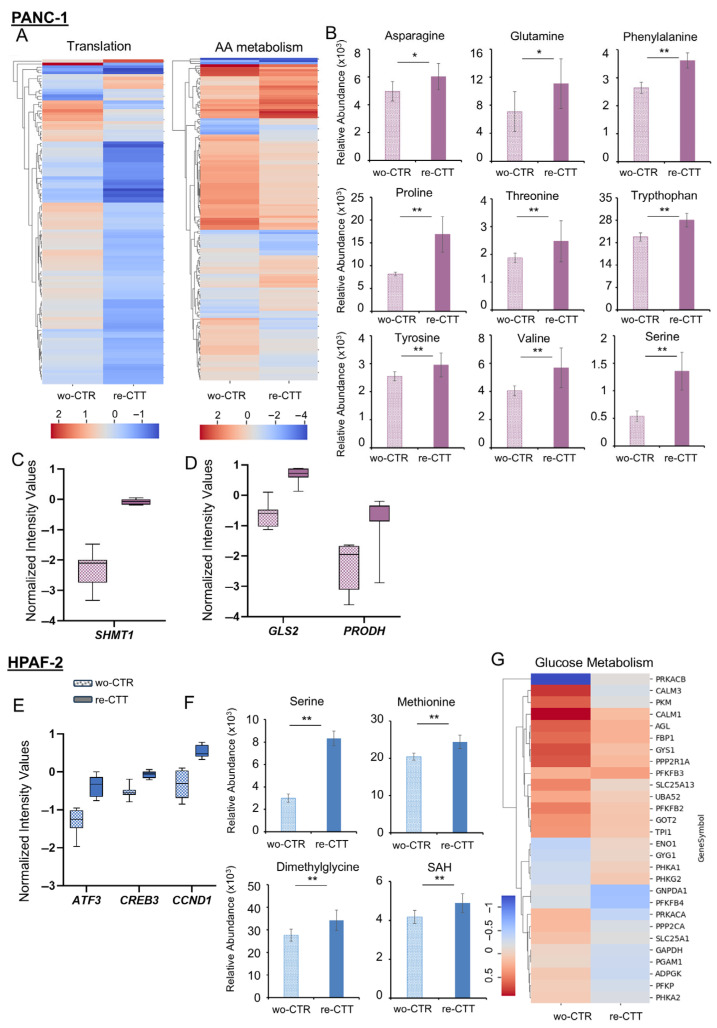
Peculiar transcriptional and metabolic rewiring for each cell line after a second round of treatment. (**A**) Hierarchical clustering of statistically significant genes involved in translation (left panel) and amino acid metabolism (right panel) in PANC-1 in wash-out control condition or in re-treatment condition obtained by microarray analysis. (**B**) Relative abundance of amino acids in PANC-1 in wash-out control condition or in re-treatment condition obtained by LC-MS. (**C**,**D**) Expression of *SHMT1* (**C**) and *GLS2* and *PRODH* (**D**) genes assessed by microarray analysis in PANC-1 wo-CTR and re-CTT. Data are expressed as normalized intensity values. (**E**) Expression of *ATF3*, *CREB3,* and *CCND1* genes assessed by microarray analysis in HPAF-2 wo-CTR and re-CTT. Data are expressed as normalized intensity values. (**F**) Relative abundance of serine, methionine, dimethylglycine, and S-adenosylhomocysteine (SAH) in HPAF-2 in wash-out control condition or in re-treatment condition obtained by LC-MS. (**G**) Hierarchical clustering of statistically significant genes involved in glucose metabolism in HPAF-2 in wash-out control condition or in re-treatment condition obtained by microarray analysis. In all panels ■ stands for erastin+methotrexate and ■ for erastin+alpelisib. Genes are represented in min to max box-and-whiskers plots. Metabolites are expressed as means ± SD. * *p* ≤ 0.05, ** *p* ≤ 0.01.

Under drug re-treatment, PANC-1 cells exhibit a mixed transcriptional and metabolic response, with both upregulated and downregulated genes within the TCA cycle and electron transport chain pathway (Figure 6C). Moreover, key TCA cycle intermediates, citrate, cis-aconitate, succinate, and malate, show increased levels in the re-CTT condition compared to wo-CTR, suggesting a metabolic shift toward enhanced oxidative metabolism (Figure 6D). Beyond these pathways, PANC-1 cells show unique metabolic adaptations, particularly in amino acid and derivative metabolism. This pathway includes translation-related genes (Figure 7A, left panel) and amino acid anabolism and catabolism genes (Figure 7A, right panel). While most translation-associated genes are downregulated in re-CTT, the upregulation of *E2F1* and *STAT1* (Figure S8A) suggests a selective transcriptional response that supports stress adaptation and cell survival rather than global suppression of translation [37,38]. Additionally, both upregulated and downregulated genes in amino acid metabolism align with the increased levels of multiple amino acids, asparagine, glutamine, phenylalanine, proline, threonine, tryptophan, tyrosine, valine, and serine, observed in re-CTT versus wo-CTR (Figure 7B). Although methotrexate disrupts nucleotide synthesis and induces metabolic stress, PANC-1 cells rewire their metabolism to compensate for this inhibition. This adaptation involves the upregulation of key metabolic and resistance-associated genes, including *SHMT1*, which compensates for methotrexate-induced folate depletion by providing essential intermediates for the nucleoside salvage pathway (Figure 7C). Upregulated genes such as *ADSS1*, *RFC2*, *TOP1*, *EXO1*, and *CDC20* (Figure S8B), along with increased levels of adenylsuccinic acid, UTP, UDP, UMP, IMP, AMP, ADP, ATP, and cAMP, may contribute to maintaining nucleotide availability (Figure S8C). *RAS* activation and cAMP signaling may also indirectly modulate growth by activating *MAPK/ERK*, *STAT3*, and *JUNB/AP-1* (Figure S8D), which promote cell cycle progression via *E2F1*, *CDC25A*, *CCNE2*, and *CCNA2* (Figure S8E), while enhancing survival and stress response through *MAPK3*, *CAMK1*, *SOS1*, and *FLOT2* (Figure S8F). Notably, *JUNB1/AP-1* can further drive *TGFB1* (Figure S8G), promoting epithelial-to-mesenchymal transition (EMT), increasing cell plasticity, survival, and resistance mechanisms [39]. These findings are further corroborated by the upregulation of *GLS2*, which enhances glutamate availability for biosynthesis and redox balance, and *PRODH*, which contributes to proline metabolism, redox homeostasis, and metabolic flexibility (Figure 7D). Additionally, the increased expression of *SLC7A3* boosts amino acid uptake, supporting metabolic demands under stress. The upregulation of *SLC27A4* and *SLC25A10*, both involved in fatty acid and mitochondrial metabolite transport, suggests an enhanced energy supply mechanism that may contribute to drug resistance (Figure S8H). Migration- and invasion-related genes such as *ITGA3* and *ITGB7*, which regulate cell adhesion, as well as *MMP11*, which modulates extracellular matrix remodeling, are also upregulated, indicating an enhanced invasive potential in re-CTT-treated PANC-1 cells (Figure S8I) [40,41,42,43]. Finally, the upregulation of *NFATC1* and *NFATC3*, a transcription factor linked to cell migration and invasion, further supports the hypothesis of increased metastatic potential under these conditions (Figure S8I) [44].

**Figure 8:**
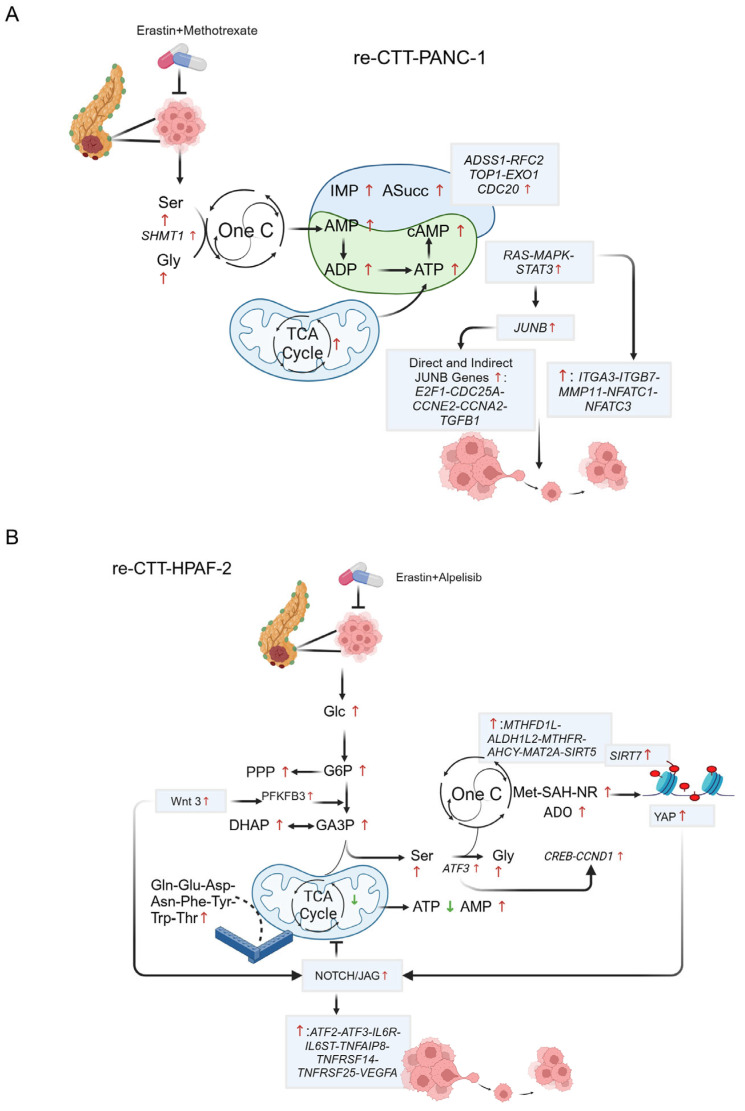
Proposed mechanism of drug resistance in PDAC cell lines. (**A**,**B**) Schematic representations of metabolites and genes involved in the resistance mechanism to double treatment in PANC-1 (**A**) and HPAF-2 (**B**). Created in https://BioRender.com.

Taken together, our data suggest that metabolic rewiring in PANC-1 re-CTT cells is driven by serine-glycine-*SHMT1*, enabling cells to bypass methotrexate treatment. *SHMT1* activation plays a key role in fueling the nucleotide salvage pathway and ensuring cAMP synthesis, which, together with *K-RAS* activation, reinforces signaling pathways (*MAPK/ERK*, *STAT3*, *JUNB/AP-1*) and increases the expression of migration- and invasion-related genes. This coordinated response, ensured by the intrinsic metabolic flexibility of PDAC cells, may enhance resistance and promote cell plasticity (Figure 8A).

#### 3.6.2. HPAF-2 Cells: Metabolic Rewiring and Inflammatory Activation in Response to Drug Re-Treatment

While in PANC-1, resistance mechanisms appear to be orchestrated by serine-glycine-*SHMT1* activation, in re-CTT-treated HPAF-2 cells, the upregulation of *ATF3*, together with the concurrent upregulation of *CREB3* and *CCND1*, suggests a coordinated regulatory strategy that promotes proliferation, survival, metastasis, and resistance. (Figure 7E). Consistently, the increased levels of serine, methionine, dimethylglycine, and S-adenosylhomocysteine (SAH) (Figure 7F), together with the upregulation of *MTHFD1L*, *ALDH1L2*, *MTHFR*, *AHCY*, and *MAT2A* (Figure S9A), indicate an enhanced flux through the folate cycle and methionine metabolism to sustain nucleotides biosynthesis (guanine, guanosine monophosphate (GMP), inosine, inosine 5′-monophosphate (IMP), xanthine, and xanthosine) and methylation-dependent epigenetic reprogramming. Increased levels of Met, SAH, and nicotinamide riboside (Figure S9B), together with the upregulation of *SIRT 5* and *SIRT 7* (Figure S9C), further suggest that resilient cells might modulate DNA and histone methylation to establish an epigenetic landscape favorable to survival [45,46,47]. Simultaneously, metabolic signature reveals a shift in glucose metabolism (Figure 7G), with the redirection of glycolytic intermediates toward the (PPP) (Figure S9B) rather than in TCA cycle activity (Figure 6C,D). This shift is further reinforced by the upregulation of *Wnt/PFKFB3* (Figure S9D) [48,49], a key glycolytic regulator that promotes glycolysis over mitochondrial respiration observed consistently with significantly decreased levels of ATP observed in re-CTT-treated HPAF-2 cells compared to wo-CTR (Figure 6D). Interesting to note, the decreased levels of TCA cycle intermediates are coupled with increased levels of amino acids, such as asparagine, aspartate, glutamate, glutamine, methionine, phenylalanine, tyrosine, tryptophan, and threonine, suggesting an adaptive response to sustain biosynthetic demands and stress-adaptive processes (Figure S9B). Moreover, the increased levels of Gln, Glu, Asp, and Asn further suggest that re-CTT-treated HPAF-2 cells might engage in glutamine anaplerosis, an alternative metabolic route to maintain bioenergetic balance and fuel biosynthetic processes.

At the transcriptional level, this metabolic adaptation is further supported by the activation of Notch signaling, as evidenced by the upregulation of *Notch2* and *JAG2* (Figure S9E). Notch activation is known to promote glycolysis while downregulating oxidative metabolism [50,51,52], further aligning with the observed metabolic rewiring. Moreover, the integration of Notch with the upregulation of *ATF3* and *ATF2* (Figure S9E), two stress-responsive transcription factors, suggests that re-CTT-treated HPAF-2 might support the orchestration of stress-responsive transcriptional programs, promoting survival under nutrient limitations and oxidative stress. These transcriptional changes are tightly linked to the activation of inflammatory pathways, as indicated by the upregulation of *IL6R*, *IL6ST*, *TNFAIP8*, *TNFRSF14*, and *TNFRSF25* (Figure S9F), which collectively promote a pro-survival inflammatory environment facilitating immune evasion and reinforcing cell viability under drug pressure. The concurrent upregulation of *VEGFA* further suggests an angiogenic response (Figure S9A), potentially enhancing nutrient acquisition and metabolic resilience in re-CTT-treated HPAF-2 cell populations.

All together, these findings reveal a well-orchestrated resistance strategy in re-CTT-treated HPAF-2 cells, in which the serine-driven metabolic rewiring appears able to bypass drug-induced metabolic constraints, ensuring sustained survival and proliferation despite repeated therapeutic exposure (Figure 8B).

## 4. Discussion

Pancreatic ductal adenocarcinoma (PDAC) remains one of the most lethal malignancies, largely due to its pronounced metabolic plasticity, which enables tumor cells to survive under adverse conditions and contributes to therapy resistance [53,54]. To maintain redox homeostasis and survive, tumor cells develop different adaptive metabolic strategies to keep ROS levels below cytotoxicity, avoiding programmed cell death [55,56]. In this context, pancreatic ductal adenocarcinoma (PDAC) cells constitute an eminent example of high-grade metabolic flexibility (Figure 1), essential for maintaining redox homeostasis. This adaptability might be implicated as a cause of the intense therapeutic resistance and poor prognosis typical of this cancer type [57,58,59]. Moving from this evidence, we deepened the metabolic rewiring underlying the aggressive phenotype of PDAC cell lines using a systems metabolomics approach. In the broader context of precision medicine, this work strengthens the idea that targeting metabolic vulnerabilities is crucial for developing more effective therapeutic strategies [60].

One of the key findings of this study is the ability of the dynamic metabolic rewiring to shape the response of PDAC cells to treatment (Figure 1). In this complex scenario, a systems biology approach allows recognition of robustness and fragility of the PDAC cell model, identifying vulnerability points for combinatorial drug treatments [61]. The identification of G6P and SAH by differential correlation analysis (DCA) as key metabolic hubs in response to erastin treatment further refines our understanding of metabolic vulnerabilities in PDAC cells. While the role of GSH in ferroptotic resistance is well-documented [62], the discovery that G6P and SAH are equally relevant in the metabolic response to treatment highlights additional targets for potential therapeutic intervention by combining erastin with alpelisib and methotrexate. If on one side, these combined treatments trigger a strong reduction in the cell growth capability in long-term proliferation curve experiments, on the other side, we discover a metabolic adaptation in which glucose-derived carbons are diverted toward alanine secretion to enhance glutamine uptake, by increased levels of glutamine transporter *ASCT2* (Figure 3) [63]. This metabolic shift toward the diversion of glutamine in several metabolic pathways (i.e., glutaminolysis, reductive carboxylation, and amino acid synthesis), particularly the unexpected redirection toward serine biosynthesis, represents a crucial adaptation to counteract oxidative stress and sustain nucleotide and amino acid metabolism [64,65,66]. These findings are aligned with previous studies demonstrating the importance of serine metabolism in tumor cell survival [67] and emphasize how PDAC cells may integrate nutrient availability to escape drug stress and activate resistance mechanisms.

A crucial implication of this work is the demonstration that metabolic adaptations extend beyond primary drug resistance to influence the response to re-treatment. The ability of PDAC cells to partially restore proliferation after combinatorial drug withdrawal underscores the dynamic nature of metabolic rewiring. The activation of different metabolic strategies shown in PANC-1 and HPAF-2 cells suggests a broader metabolic rewiring that integrates gene adaptation and stress-responsive pathways with metabolic resilience [68,69]. It is interesting to note that while PANC-1 exhibits a preference for oxidative metabolism under treatment, HPAF-2 engages inflammatory pathways or increases glycolytic flux. This metabolic heterogeneity highlights the importance of carefully considering it when designing therapeutic approaches [70]. Furthermore, the observation that *SHMT1*, Notch, and inflammatory cytokine activation contribute to therapy resistance in our PDAC cell lines suggests that a combination of metabolic inhibitors and immunomodulatory approaches may be needed to fully counteract PDAC’s adaptability [50,71,72,73].

Collectively, our findings enhance the current understanding of PDAC metabolic resilience where treatment-induced stress elicits a compensatory response able to partially restore proliferation. The ability of PDAC cells to dynamically rewire metabolism, especially through the serine synthesis pathway, represents a key vulnerability that could be exploited in future therapeutic strategies. Targeting these metabolic adaptations, possibly in combination with ferroptosis inducers or immune modulators, holds promise for overcoming therapy resistance and improving patient outcomes. Future studies should focus on integrating metabolic inhibitors with existing treatments to assess their efficacy in pre-clinical and clinical settings, ultimately paving the way for more effective interventions against this highly aggressive cancer [3,74,75].

## 5. Conclusions

Pancreatic cancer is characterized by profound metabolic plasticity, which enables tumor cells to survive under therapeutic stress and contributes to treatment failure. In this study, we demonstrate that such metabolic adaptability is not merely a passive hallmark but an active, stress-induced survival strategy that rewires core metabolic pathways in response to combinatorial treatment. By integrating untargeted metabolomics, stable isotope tracing, and transcriptomics across multiple pancreatic cancer cell lines, we reveal that combination treatments targeting redox homeostasis and metabolic pathways initially suppress growth but ultimately trigger a metabolic rebound. A central and unexpected finding is the activation of serine metabolism, via both de novo synthesis and enhanced uptake, as a key mechanism of adaptive resilience. This serine-driven response supports redox balance, nucleotide biosynthesis, and cell survival, representing a metabolic signature of resilience to combinatorial drug treatment. Moreover, our data show that this adaptation is coupled with transcriptional programs promoting proliferation, migration, and inflammatory signaling, further contributing to therapeutic tolerance. These findings advance our understanding of pancreatic cancer by identifying serine metabolism as a dynamic biomarker of treatment adaptation and a promising therapeutic target. Importantly, the systems-level approach employed here enables the discovery of functional metabolic networks, offering a translational framework for the development of personalized, metabolism-based treatment therapies. Future studies will be essential to validate serine metabolism as a functional biomarker of adaptive response and resistance mechanisms, with potential implications for treatment stratification in pancreatic cancer.

## Data Availability

Transcriptional data presented in this work have been deposited in the National Center for Biotechnology Information Gene Expression Omnibus (GEO) (https://www.ncbi.nlm.nih.gov/geo/, accessed on 25 March 2025) and are accessible through GEO Series accession numbers GSE292890 and GSE292891. All other data will be made available on reasonable request.

## Supplementary Materials

The following supporting information can be downloaded at https://www.mdpi.com/article/10.3390/antiox14070833/s1. Figure S1: Proliferation, metabolic profiling, and respiratory capacity of PDAC cell lines. (A) Proliferation curves of eight PDAC cancer cell lines in normal growth condition (**―**), in low glutamine medium (**―**), or low glucose medium (**―**). Cells were grown in 6-well plates in the appropriate growth medium. Cells were collected and counted at the indicated time points. Error bars indicate SD. (B) Hierarchical clustering heatmaps showing significantly different intracellular metabolites in all PDAC cell lines as detected by LC-MS. Colors represent different levels that increase from blue to red. (C) Mitochondrial respiration reflected by OCR levels assessed by Mitostress Seahorse analysis under basal conditions or following the addition of oligomycin (1 μM), the uncoupler FCCP (1 μM), or the electron transport inhibitor rotenone (0.5 μM). Analysis was conducted on eight PDAC cell lines in normal growth conditions (n = 5). (D) Mitochondrial basal and spare respiration reflected by OCR levels assessed by Mitostress Seahorse analysis on eight PDAC cell lines in normal growth condition (n = 5). Figure S2: Erastin scheme of action, cell viability, and lipid peroxidation in treated PDAC cell lines. (A) Graphical representation of xCT transporter and its inhibition by pharmacological treatment by erastin. (B) Cell viability of eight PDAC cell lines after erastin treatment for 48 h at the indicated concentration. Data are expressed as relative to the control. (C) Lipid peroxidation levels assessed by detection of malondialdehyde (MDA) with the Lipid Peroxidation (MDA) Assay Kit in eight PDAC cell lines in normal growth conditions or after erastin treatment (n = 5). All data are expressed as means ± SD. * *p* ≤ 0.05, ** *p* ≤ 0.01. Figure S3: Respiratory capacity of PDAC cell lines under erastin treatment: mitochondrial respiration (on the left) and the relative mitochondrial basal, maximal and spare respiration, and ATP production reflected by OCR levels assessed by Mitostress Seahorse analysis on eight PDAC cell lines in normal growth condition or after erastin treatment (n = 10). All data are expressed as means ± SD. * *p* ≤ 0.05. Figure S4: Proliferation with erastin of PDAC cell lines. Proliferation curves of eight PDAC cancer cell lines in normal growth conditions (**―**) or under erastin treatment (**―**) at the indicated concentrations (IC_50_ at 48 h for each cell line). Cells were collected and counted at the indicated time points. Figure S5: Cell viability in treated PDAC cell lines. (A–D) Cell viability of eight PDAC cell lines after alpelisib (A), everolimus (B), resveratrol (C), and methotrexate (D) treatment for 48 h at the indicated concentration. Data are expressed as relative to the control. Figure S6: Molecular and metabolic characterization of PDAC cell lines after combinatorial treatment. (A) Alanine secretion in the extracellular medium of Panc 10.05 and Capan-2 (lower panel) in the presence or the absence of combinatorial treatment based on relative abundance obtained by GC-MS. Alanine secretion is calculated as the difference between alanine in the spent medium after 48 h and alanine in the medium at t = 0. (B) Extracellular glutamine uptake and glutamate secretion determined enzymatically using YSI2950 bioanalyzer in Panc 10.05 and Capan-2 in the presence or the absence of combinatorial treatment. (C) Relative glutamine abundance in Panc 10.05 and Capan-2 in normal growth conditions or in the presence of combinatorial obtained by LC-MS. (D) Relative glutamate, proline, asparagine, serine, and glycine abundance in Panc 10.05 and Capan-2 in normal growth conditions or in the presence of combinatorial obtained by LC-MS. (E) *ASCT2* gene expression assessed by real-time qPCR in Panc 10.05 and Capan-2 cell lines after combinatorial treatment. (F) Expression of genes related to de novo serine synthesis pathway assessed by real-time qPCR in Panc 10.05 and Capan-2 cell lines after combinatorial treatment. The molecular data were normalized to β-actin, and the ΔΔct values were expressed as the fold of induction (FOI) of the ratio between treated and control cells. (G) Expression of genes related to anabolic processes assessed by real-time qPCR in PANC-1, HPAF-2, Panc 10.05, and Capan-2 cell lines after combinatorial treatment. The molecular data were normalized to β-actin, and the ΔΔct values were expressed as the fold of induction (FOI) of the ratio between treated and control cells. (H) Expression of Nrf2, KEAP, and HIF-1α assessed by real-time qPCR in Panc 10.05 cell line after combinatorial treatment. The molecular data were normalized to β-actin, and the ΔΔct values were expressed as the fold of induction (FOI) of the ratio between treated and control cells. (I) Total ROS levels measured by DCFDA staining in Panc 10.05 in normal growth condition or in the presence of combinatorial treatment. (J) Autophagy levels assessed by CYTO-ID^®^ Autophagy detection kit in Panc 10.05 in normal growth condition or in the presence of combinatorial treatment. (K) NAD^+^/NADH ratio in PANC-1and HPAF-2 in wash-out control condition or in re-treatment condition obtained by LC-MS. Figure S7: Integration of transcriptomics and metabolomics data. (A,B) Bar plot for PANC-1 (A) and HPAF-2 (B) of pathway enrichment of the integrated transcriptomics–metabolomics analysis. Over-representation analysis was performed using the statistically significant lists of genes and metabolites obtained from the two different analyses using Reactome pathways as functional database. Figure S8: Molecular and metabolic characterization of PANC-1 cell line after a second round of treatment. (A,B,D,E,F,G,H,I) Expression of indicated genes assessed by microarray analysis in PANC-1 wo-CTR and re-CTT. Data are expressed as normalized intensity values and represented as min to max box-and-whiskers plots. (C) Hierarchical clustering heatmaps showing significantly different intracellular metabolites in PANC-1 wo-CTR and re-CTT as detected by LC-MS. Colors represent different levels that increase from blue to red. Figure S9: Molecular and metabolic characterization of HPAF-2 cell line after a second round of treatment. (A,C,D–G) Expression of indicated genes assessed by microarray analysis in HPAF-2 wo-CTR and re-CTT. Data are expressed as normalized intensity values and represented as min to max box-and-whiskers plots. (B) Hierarchical clustering heatmaps showing significantly different intracellular metabolites in HPAF-2 wo-CTR and re-CTT as detected by LC-MS. Colors represent different levels that increase from blue to red. Supplementary Table S1: List of primers used for real-time PCR. Table S2: List of xCT inhibitors and mechanism of action. Supplementary File S1: List of statistically significant genes and their expression values. The different Excel sheets are relative to the genes used for the creation of the heatmaps presented in Figure 6C and  Figure 7A,G.

## Abbreviations

The following abbreviations are used in this manuscript:

2-AmAd	L-2-Aminoadipic acid
2OxB	2-oxobutanoate
3PG	3-Phosphoglyceric acid
6PG	6-phosphogluconic acid
ACLY	ATP citrate lyase
Ade	Adenine
Ado	Adenosine
ADP	Adenosine diphosphate
Akg	α-Ketoglutaric acid
Ala	L-Alanine
ALT	Aminotransferases
ANOVA	Analysis of variance
AMP	Adenosine monophosphate
ArgSucc	Arginosuccinic acid
Asn	L-Asparagine
Asp	L-aspartic acid
ATP	Adenosine triphosphate
BHT	Butylated hydroxytoluene
cAMP	Cyclic AMP
CCK-8	Cell Counting Kit-8
CDP	Cytidine diphosphate
Cis-Aco	Cis Aconitic acid
Cit	Citric acid
CO_2_	Carbon dioxide
CT	Citrulline
CTR	Control
CTT	Combinatorial treatment
Cy3	Cyanine3
Cys	L-Cysteine
Cys-Cys	L-Cystine
DCA	Differential correlation analysis
DCFDA	Dichloro-dihydro-fluoresceine-diacetate
DHAP	Dihydroxyacetone phosphate
DMEM	Dulbecco’s Modified Eagle’s Medium
E4P	D-Erythrose-4-phosphate
ER	Erastin
F1,6BP	Fructose 1,6 bisphosphate
FBS	Fetal Bovine Serum
FCCP	Carbonyl cyanide p-(trifluoromethoxy)phenylhydrazone
FDR	False discovery rate
FOI	Fold of induction
Fum	Fumaric acid
G3P	Glyceraldehyde 3-phosphate
G6P/Glc6P	Glucose 6-phosphate
GC-MS	Gas chromatography–mass spectrometry
GDH	Glutamate dehydrogenase
GDP-Glc	GDP-glucose
Glc	Glucose
Gln	L-Glutamine
GLS	Glutaminase
Glt	Glutaric acid
Glu	L-Glutamic acid
Glu5P	L-Glutamic acid 5-phosphate
Gly	Glycine
Glyc3P	Glycerol 3-phosphate
GMP	Guanosine monophosphate
GOA	Glyoxylic acid
GOT1/GOT2	Glutamic oxaloacetic transaminases
GSH	Reduced glutathione
GSSG	Oxidized glutathione
H6P	Hexose 6-phosphate
HBP	Hexosamine biosynthesis pathway
HIF1α	Hypoxia Inducible Factor 1 Subunit Alpha
Hpx	Hypoxanthine
ICit	Isocitric acid
IMP	Inosine 5′-monophosphate
Ino	Inosine
Lac	Lactic acid
LC-MS	Liquid chromatography–mass spectrometry
LDH	Lactate dehydrogenase
Leu	L-Leucine
Mal	Malic acid
MDA	Malondialdehyde
MDH1	Malate dehydrogenase
ME1	Malic enzyme
Met	L-Methionine
MID	Mass Isotopologue Distribution
MTBSTFA	N-tert-Butyldimethylsilyl-N-methyltrifluoroacetamide
mTOR	Mammalian target of rapamycin
MTX	Methotrexate
N-AcGlc	N-Acetylglucosammine
N-AcGlc6P	N-Acetylglucosammine 6-phosphate
NAD	Oxidized nicotinammide adenine dinucleotide
NADH	Reduced nicotinammide adenine dinucleotide
NADP	Oxidized nicotinammide adenine dinucleotide phosphate
NADPH	Reduced nicotinammide adenine dinucleotide phosphate
NEAA	Non-essential amino acids
OAA	Oxalacetic acid
OCR	Oxygen consumption rate
Orn	Ornithine
PBS	Phosphate-buffered saline
PC	Pyruvate carboxylase
PDAC	Pancreatic ductal adenocarcinoma
PDH	Pyruvate dehydrogenase
PEP	Phosphoenolpyruvate
PFP	Pentafluorophenyl
Phe	L-Phenylalanine
PI3K	Phosphatidylinositol-3 kinase
PKM2	Pyruvate kinase M2 subtype
PPP	Pentose phosphate pathway
Pro	L-Proline
Pyr	Pyruvic acid
PyrGlu	Pyroglutamic acid
QTOF	Quadrupole time-of-flight
RedB2	Reduced riboflavin
RIN	RNA integrity number
ROS	Reactive oxygen species
RPMI	Roswell Park Memorial Institute
Ru5P	Ribulose 5-phosphate
S7P	Sedoheptulose 7-phosphate
SAH	S-Adenosylhomocysteine
SAICAR	1-(phosphoribosyl)imidazolecarboxamide
sCoA	Succinyl-CoA
Ser	L-Serine
SS	Succinyl-CoA synthetase
SSP	Serine synthesis pathway
Succ	Succinic acid
TBDMCS	tert-Butyldimethylchlorosilane
TCA	Tricarboxylic acid
Thr	L-Threonine
Trp	L-Triptophan
Tyr	L-Tyrosine
UAC	Uric acid
UDP-Glc	UDP-Glucose
UDP-N-AcGlc	UDP-N-Acetylglucosammine
UHPLC	Ultra-High pressure liquid chromatography
UDP	Uridine diphosphate
UMP	Uridine monophosphate
UTP	Uridine triphosphate
Val	L-Valine
VDAC	Voltage-dependent anion channel
Xyl5P	Xylulose 5-phosphate
γ-Glu_Cys	γ-glutamylcysteine
ΔΨm	Mitochondrial membrane potential
