# Supplementary material for: Serine-Driven Metabolic Plasticity Drives Adaptive Resilience in Pancreatic Cancer Cells
Source: Antioxidants (Basel). 2025 Jul 7;14(7):833. doi: 10.3390/antiox14070833 (PMC12291976; doi:10.3390/antiox14070833)
Supplement: Supplementary file 1 [file antioxidants-14-00833-s001.zip › Supplementary Table S2.pdf]

Supplementary Table S1. Erastin shows combinatorial effectiveness metabolic pathways.

Drugs	Primary Mechanism of Induction	Effects on Mitochondria
Erastin	Inhibits system Xc^-, depletes glutathione (GSH), leads to lipid peroxidation and ferroptosis.	Directly affects mitochondrial function by interacting with VDACs, leading to mitochondrial membrane potential loss and ROS production.
Sorafenib	Multi-kinase inhibitor; indirectly induces ferroptosis by reducing GSH and inhibiting system Xc^-.	Partially affects mitochondrial function. Can cause mitochondrial stress via MAPK pathway inhibition, but not a direct VDAC interactor like Erastin.
Sulfasalazine	Inhibits system Xc^-, reducing GSH levels and inducing ferroptosis.	Minimal direct effect – Its main mechanism involves oxidative stress through cystine deprivation, rather than mitochondrial impairment.

Although Erastin is not yet clinically approved, its unique ability to inhibit both system Xc^- and mitochondrial function makes it an ideal compound for studying metabolic shifts, including those associated with ferroptosis mechanisms. Therefore, Erastin was specifically chosen for its dual role in simultaneously inhibiting system Xc^- and mitochondrial activity, a mechanism that could be targeted with clinically relevant agents in future studies, potentially guiding the optimization of FDA-approved ferroptosis inducers such as Sorafenib or Sulfasalazine for PDAC treatment
