# Supplementary figures and images for: Serine-Driven Metabolic Plasticity Drives Adaptive Resilience in Pancreatic Cancer Cells
Source: Antioxidants (Basel). 2025 Jul 7;14(7):833. doi: 10.3390/antiox14070833 (PMC12291976; doi:10.3390/antiox14070833)

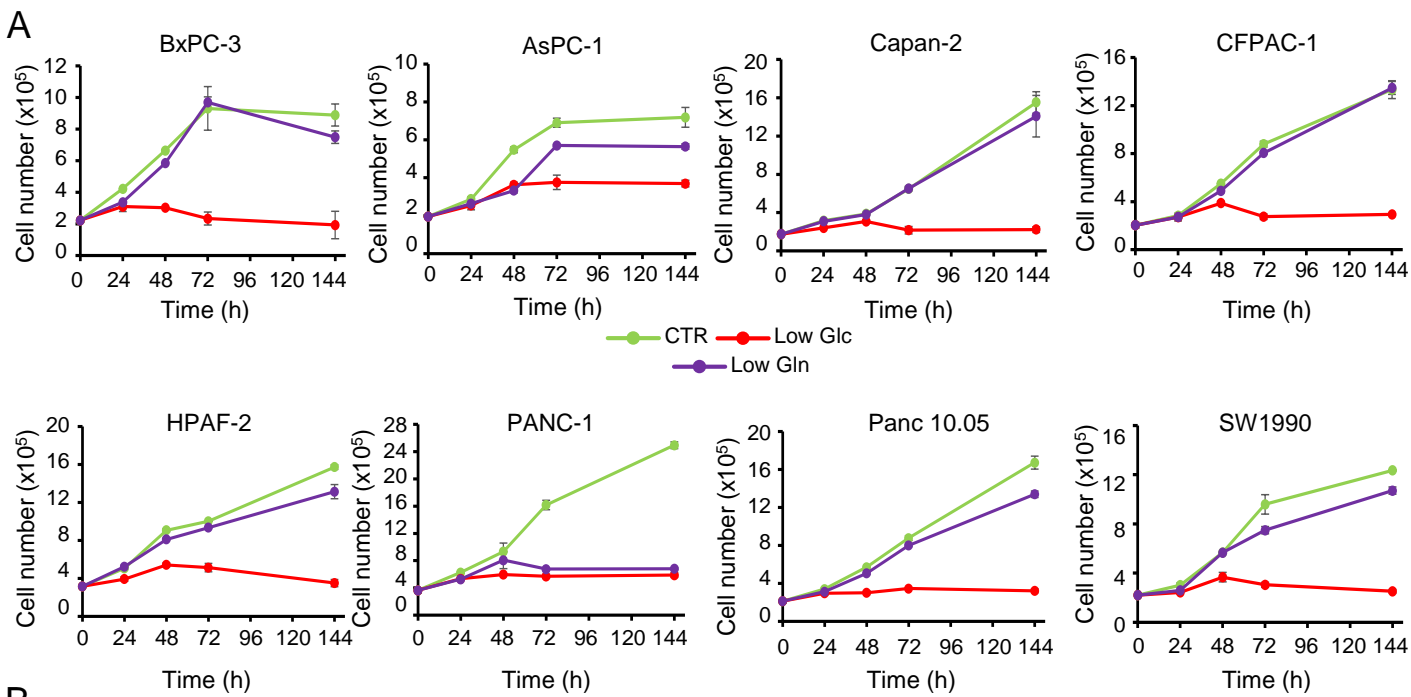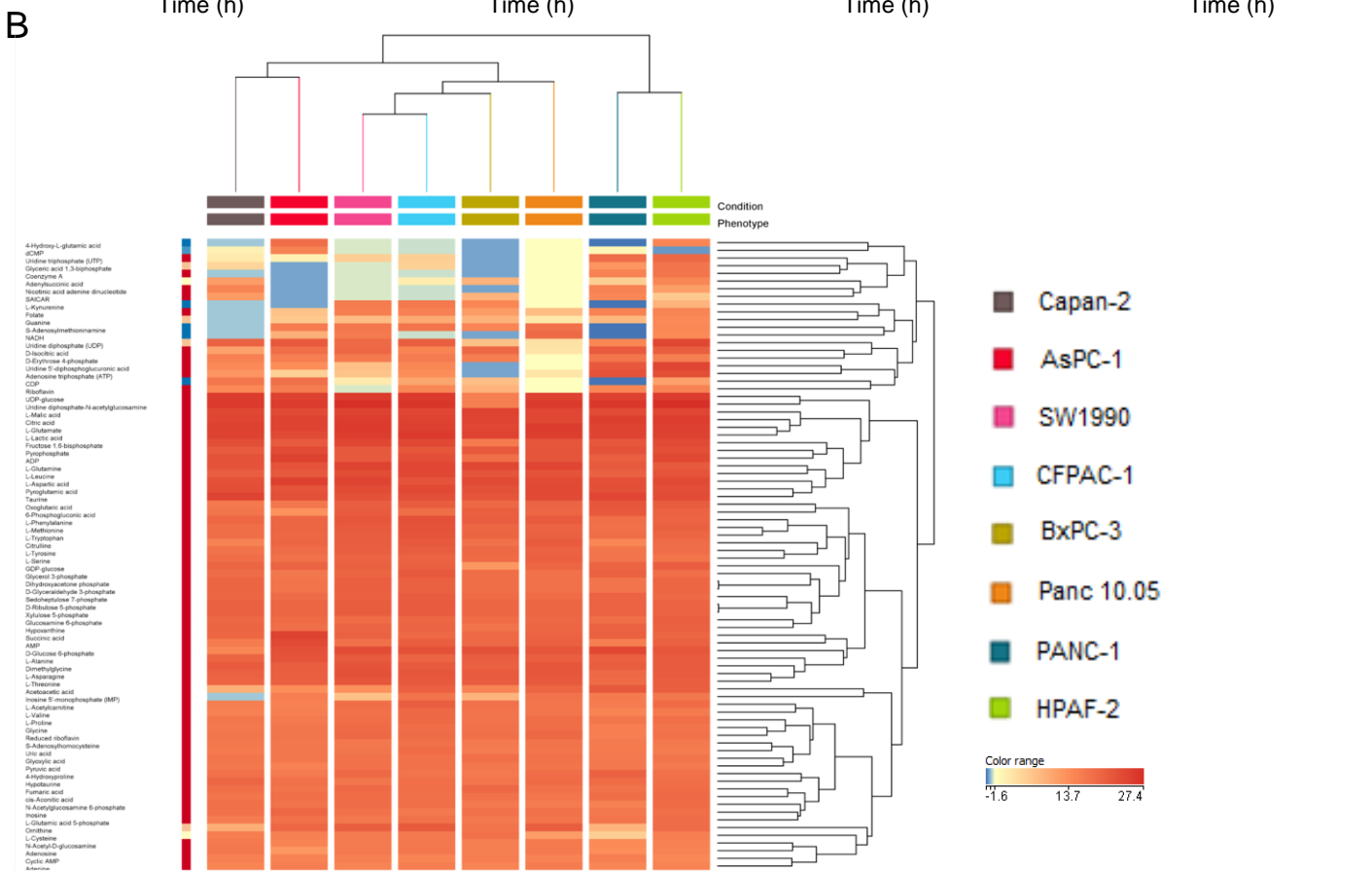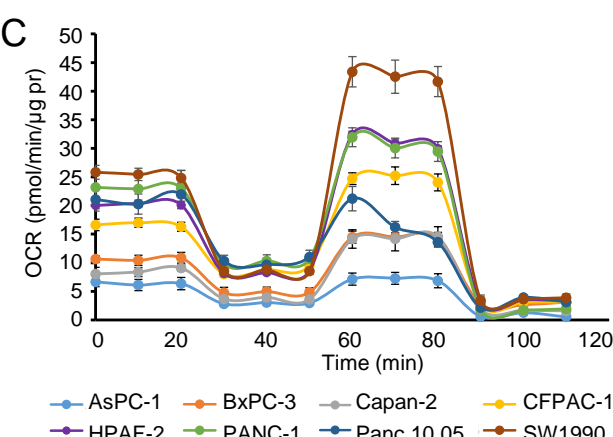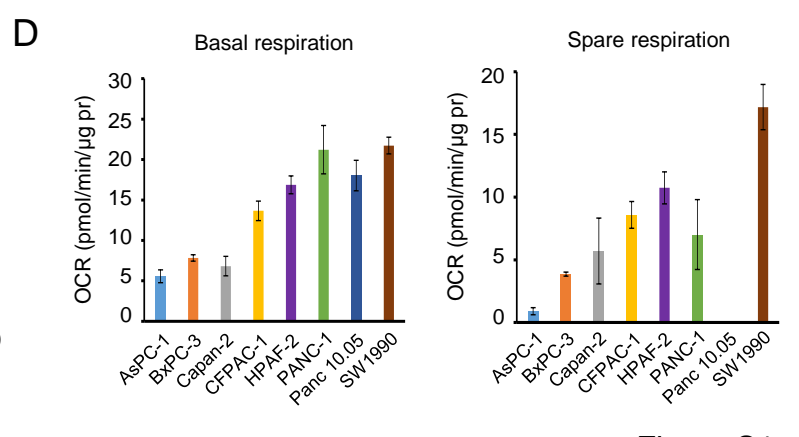

Figure S1

A

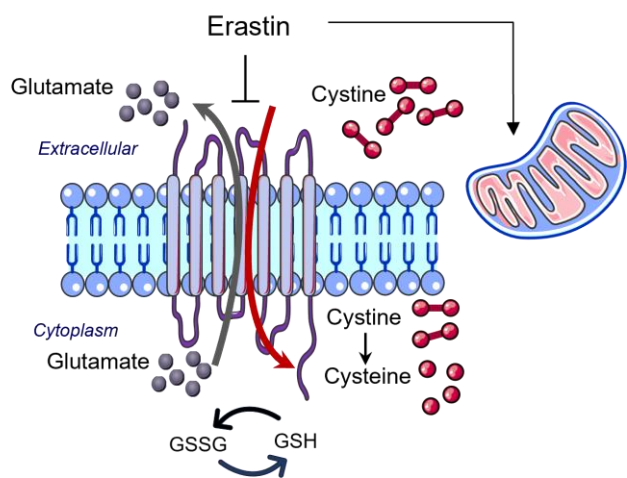

B

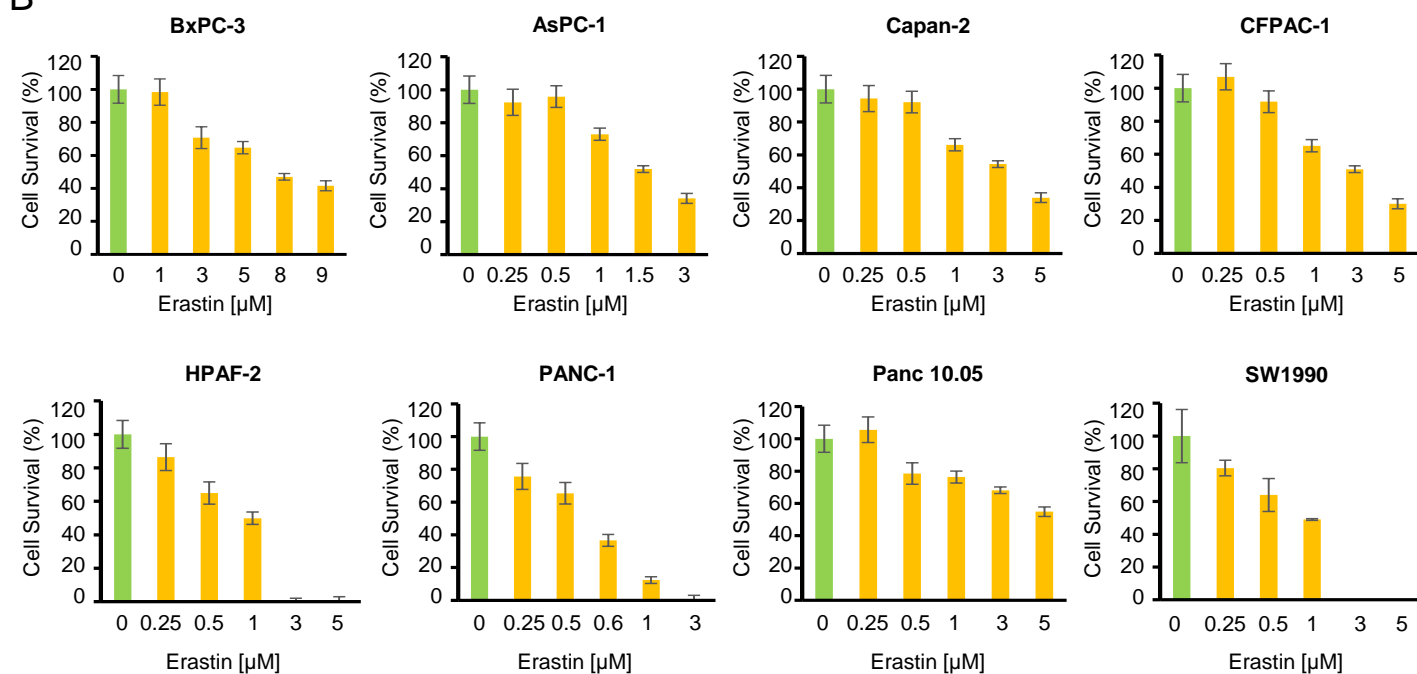

C

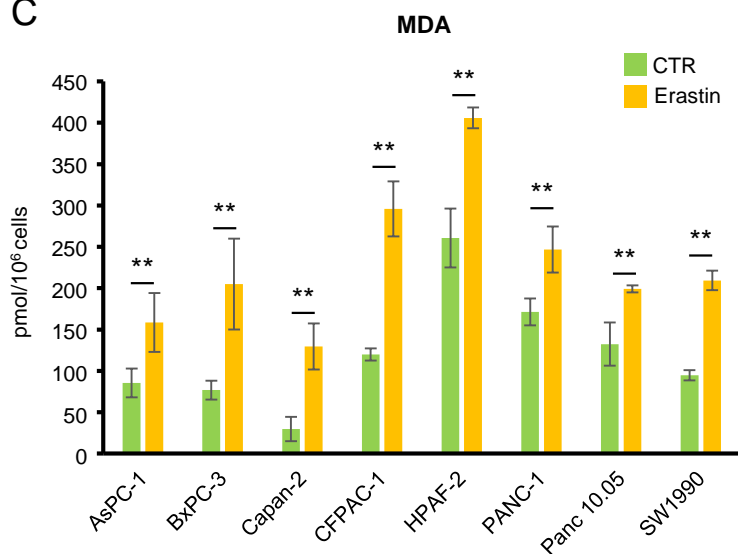

Figure S2

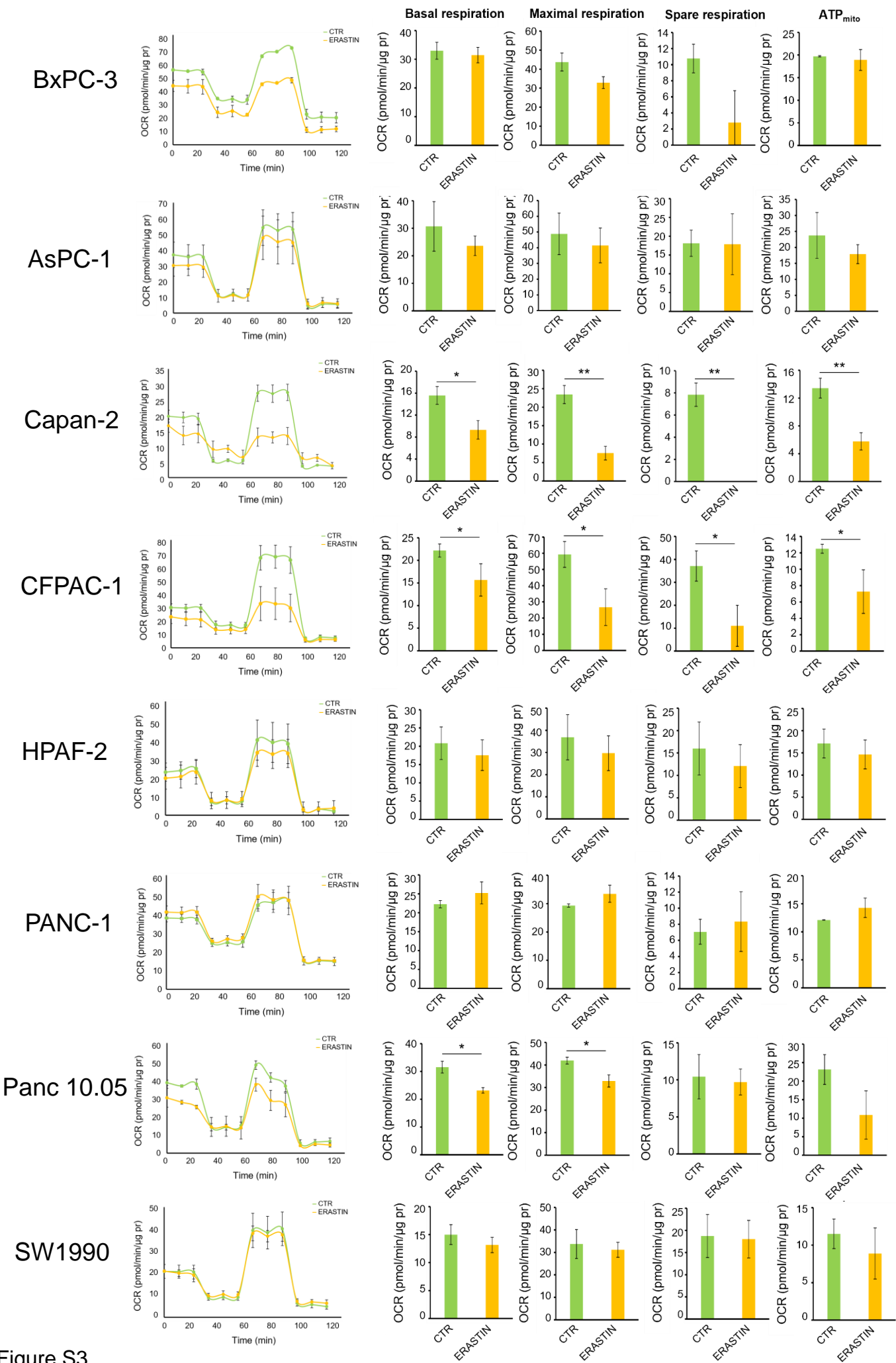

Figure S3

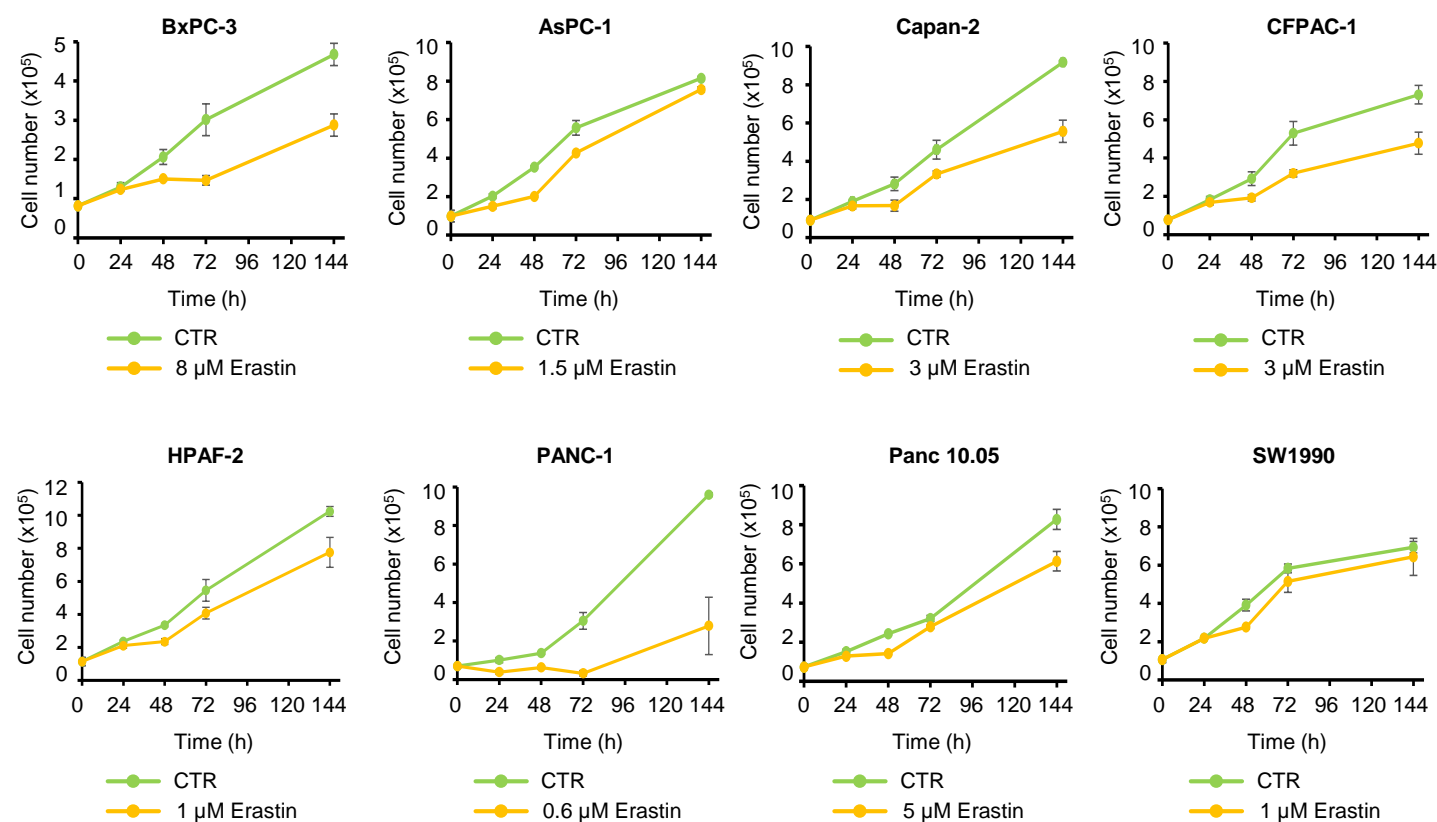

Figure S4

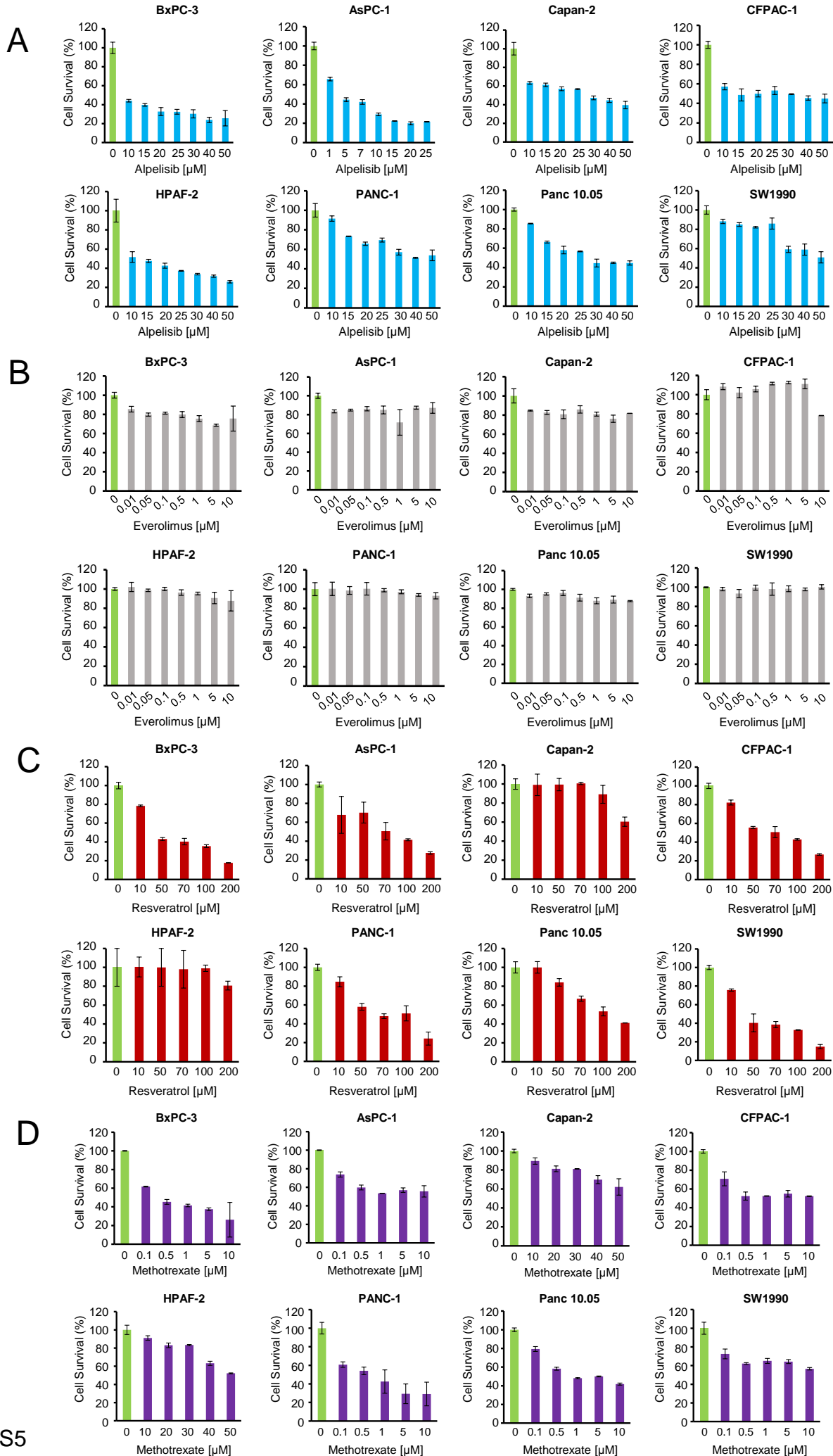

Figure S5

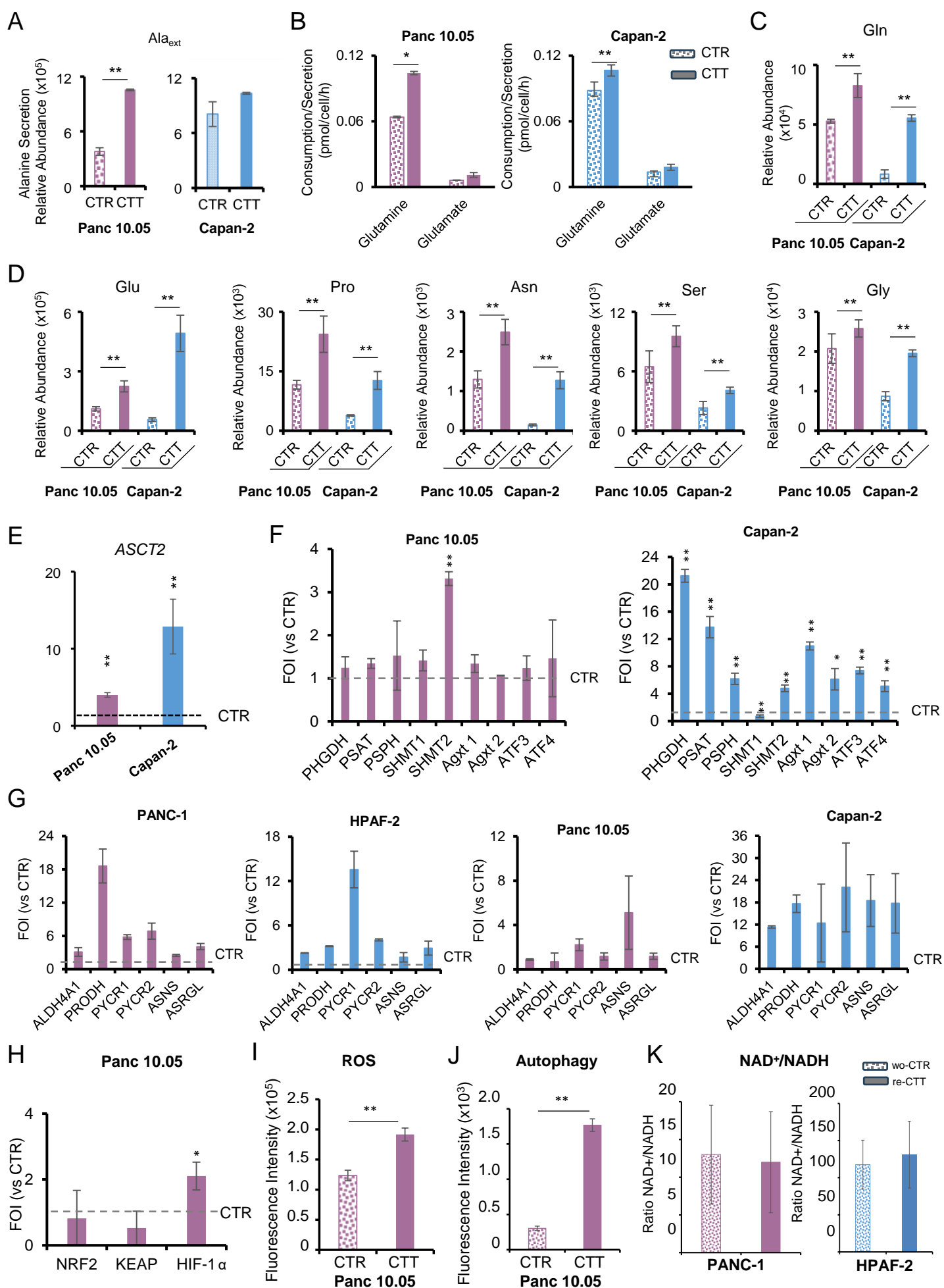

Figure S6

A

PANC-1

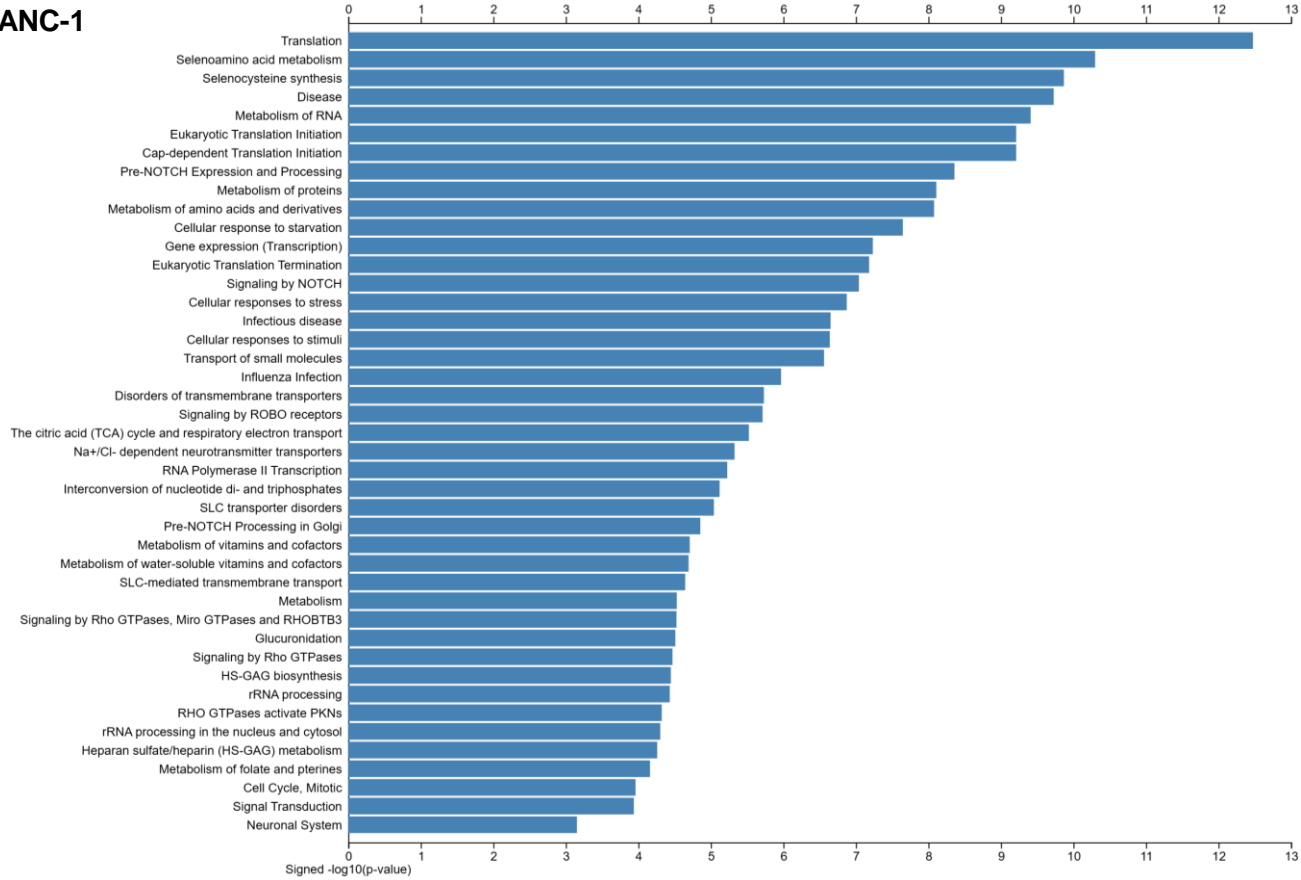

B

HPAF-2

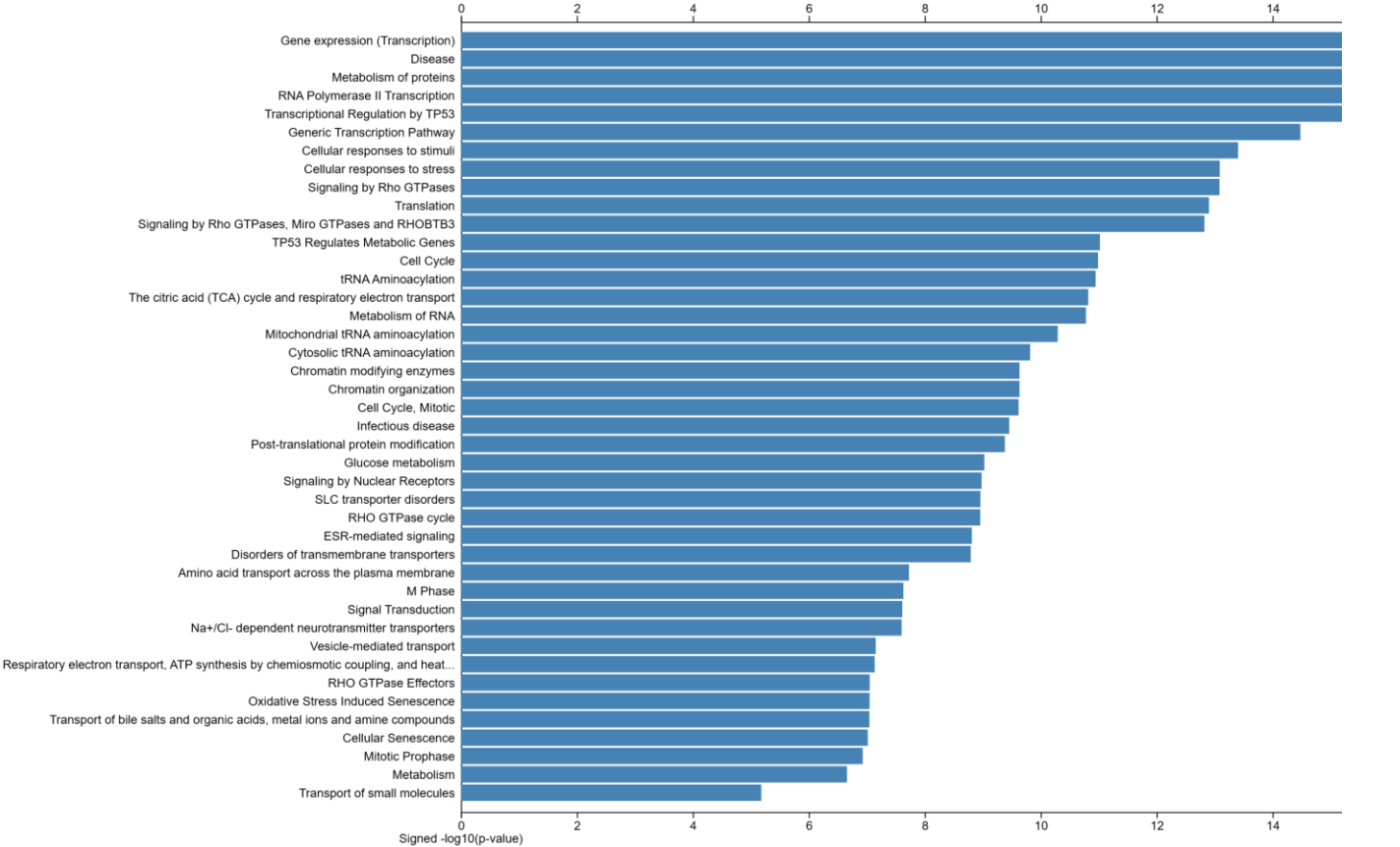

Figure S7

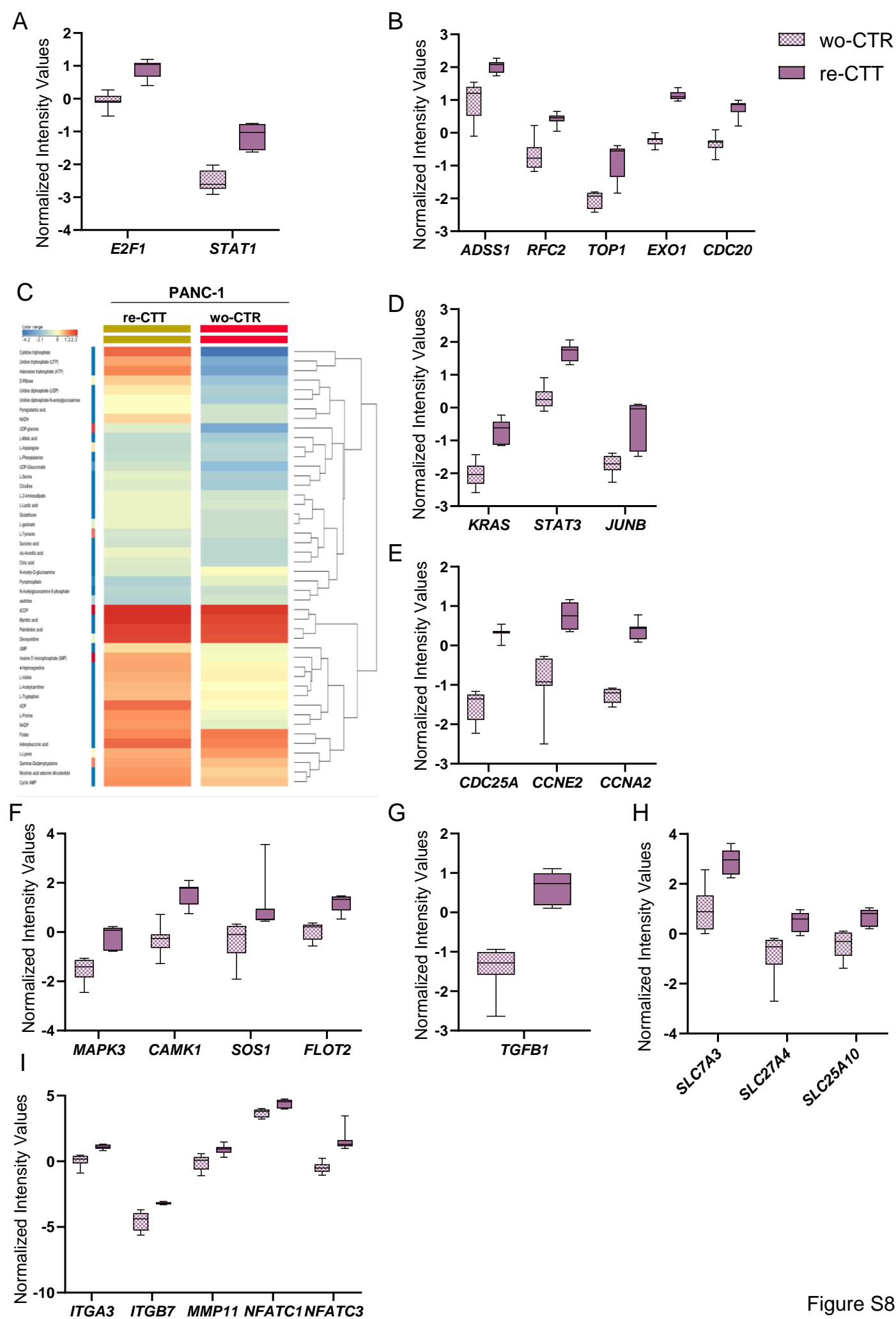

Figure S8

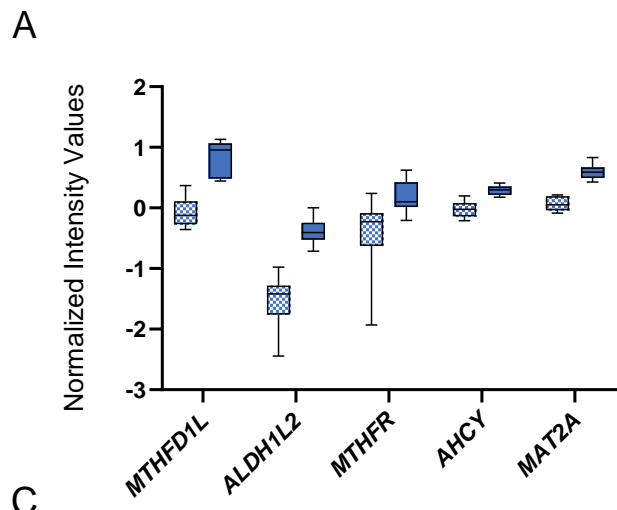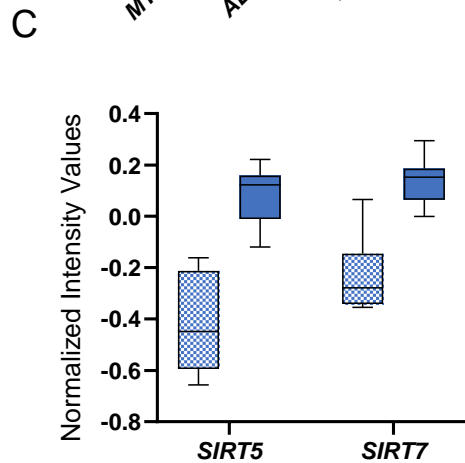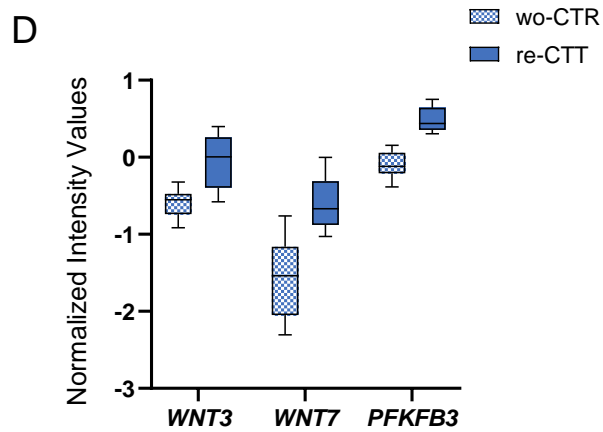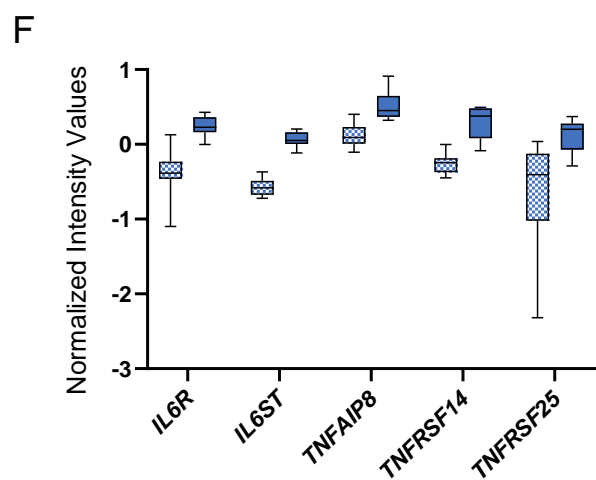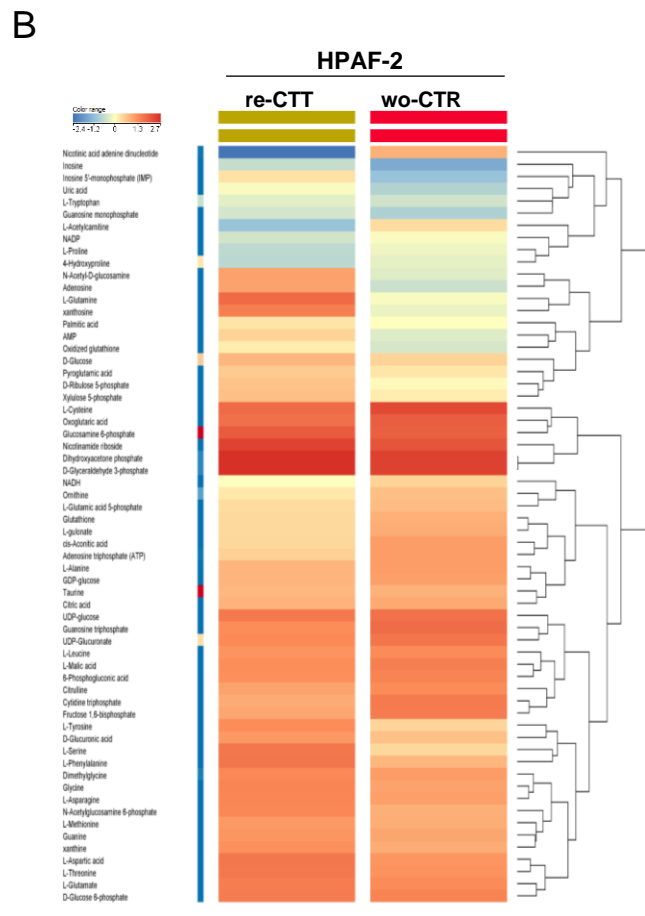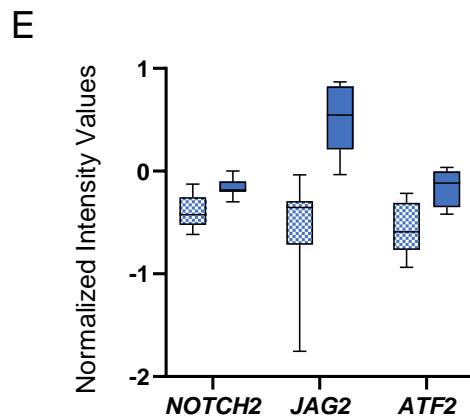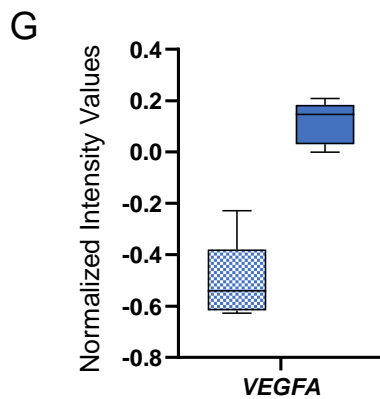

Figure S9

Supplement: Supplementary file 1 [file antioxidants-14-00833-s001.zip › Supplementary Figures.pdf]
